# Integrative omics analysis reveals insights into small colony variants of *Staphylococcus aureus* induced by sulfamethoxazole-trimethoprim

**DOI:** 10.1186/s12866-024-03364-8

**Published:** 2024-06-14

**Authors:** Jingwen Zhou, Chunyan He, Han Yang, Wen Shu, Qingzhong Liu

**Affiliations:** 1 Department of Clinical Laboratory, Shanghai Municipal Hospital of Traditional Chinese Medicine, Shanghai University of Traditional Chinese Medicine, 274 Zhijiang Middle Rd, Shanghai, 200071 People’s Republic of China; 2 Department of Clinical Laboratory, Shanghai General Hospital, Shanghai Jiaotong University School of Medicine, Shanghai, China

**Keywords:** Small colony variant, *Staphylococcus aureus*, Genomics, Transcriptomics, Metabolomics, Sulfamethoxazole-trimethoprim

## Abstract

**Background:**

Long-term treatment with trimethoprim-sulfamethoxazole (SXT) can lead to the formation of small-colony variants (SCVs) of *Staphylococcus aureus*. However, the mechanism behind SCVs formation remains poorly understood. In this study, we explored the phenotype and omics-based characterization of *S. aureus* SCVs induced by SXT and shed light on the potential causes of SCV formation.

**Methods:**

Stable SCVs were obtained by continuously treating *S. aureus* isolates using 12/238 µg/ml of SXT, characterized by growth kinetics, antibiotic susceptibility testing, and auxotrophism test. Subsequently, a pair of representative strains (SCV and its parental strain) were selected for genomic, transcriptomic and metabolomic analysis.

**Results:**

Three stable *S. aureus* SCVs were successfully screened and proven to be homologous to their corresponding parental strains. Phenotypic tests showed that all SCVs were non-classical mechanisms associated with impaired utilization of menadione, heme and thymine, and exhibited slower growth and higher antibiotic minimum inhibitory concentrations (MICs), compared to their corresponding parental strains. Genomic data revealed 15 missense mutations in 13 genes in the representative SCV, which were involved in adhesion, intramolecular phosphate transfer on ribose, transport pathways, and phage-encoded proteins. The combination analysis of transcriptome and metabolome identified 35 overlapping pathways possible associated with the phenotype switching of *S. aureus*. These pathways mainly included changes in metabolism, such as purine metabolism, pyruvate metabolism, amino acid metabolism, and ABC transporters, which could play a crucial role in promoting SCVs development by affecting nucleic acid synthesis and energy metabolism in bacteria.

**Conclusion:**

This study provides profound insights into the causes of *S. aureus* SCV formation induced by SXT. The findings may offer valuable clues for developing new strategies to combat *S. aureus* SCV infections.

**Supplementary Information:**

The online version contains supplementary material available at 10.1186/s12866-024-03364-8.

## Background

*Staphylococcus aureus* is a significant bacterium causing a wide range of diseases, from mild skin infections to severe invasive infections [1]. The prevalence of multidrug-resistant *S. aureus* poses challenges for treatment with β-lactam drugs, leading to an increased importance of non-β-lactam antibacterial agents like trimethoprim-sulfamethoxazole (SXT) and doxycycline [2]. SXT has been reported to be effective against *S*. *aureus*, and is considered a valuable drug for treating *S*. *aureus* infections [3–6]. However, prolonged treatment with SXT can lead to the formation of small-colony variants (SCVs) in *S. aureus* [3].

SCVs are characterized by impaired growth, reduced virulence, and increased resistance to antibiotics [7]. SCVs enhance the ability of bacteria to survive within host cells, allowing them to evade the immune system and antibiotics [8], and are often associated with recurrent and chronic refractory infections [7, 9, 10]. The switching of bacteria from wild cell-type to SCVs phenotype involves significant changes in the metabolism, gene mutations, or expression of virulence genes [7]. However, little is known about the specific differences in SXT-associated *S. aureus* SCVs.

In this study, we induced stable SCVs by using SXT to treat clinical *S. aureus* strains in vitro. We then explored the possible internal changes of the pathogen during the phenotypic switching using multi-omics methods (genomic, transcriptomic, and metabolomic), which might provide clues for the development of new strategies to control *S. aureus* SCVs infections.

## Results

### SXT-induced stable phenotype of *S. aureus* SCVs

**Fig. 1:**
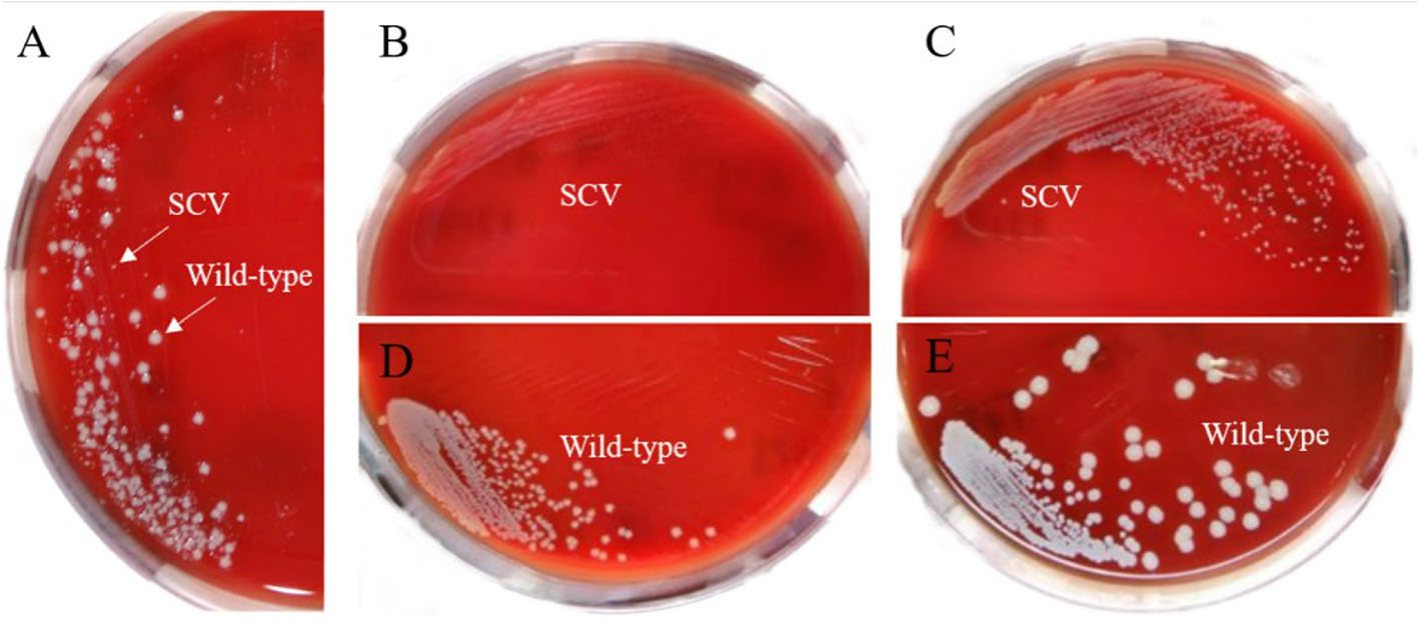
Phenotype of wild-type and small-colony variant (SCV) originated from representative strain 15 of *S*. *aureus*. **A** SCV phenotype observed after 15 days of induction with 12/238 µg/ml of SXT, cultured for 24 h. **B** SCV cultured for 24 h after screening through ≥ 10 passages of subculture. **C** SCV cultured for 48 h after screening through ≥ 10 passages of subculture. **D** Wild-type parental strain cultured for 24 h. **E** Wild-type parental strain cultured for 48 h

Among the 30 clinical wild-type isolates, 3 strains (2, 15 and 29) exhibited the SCVs phenotype after 11, 15 and 5 days of subculturing with SXT, respectively (Fig. 1). The induced small colonies were less than one-tenth the size of the colonies related to their parental strains and exhibited slower growth. Even after 10 passages of subculture on antibiotic-free plates, the SCVs maintained the small colony size, indicating their stability as a distinct cell type.

### Identification and genotyping of SCVs

**Fig. 2:**
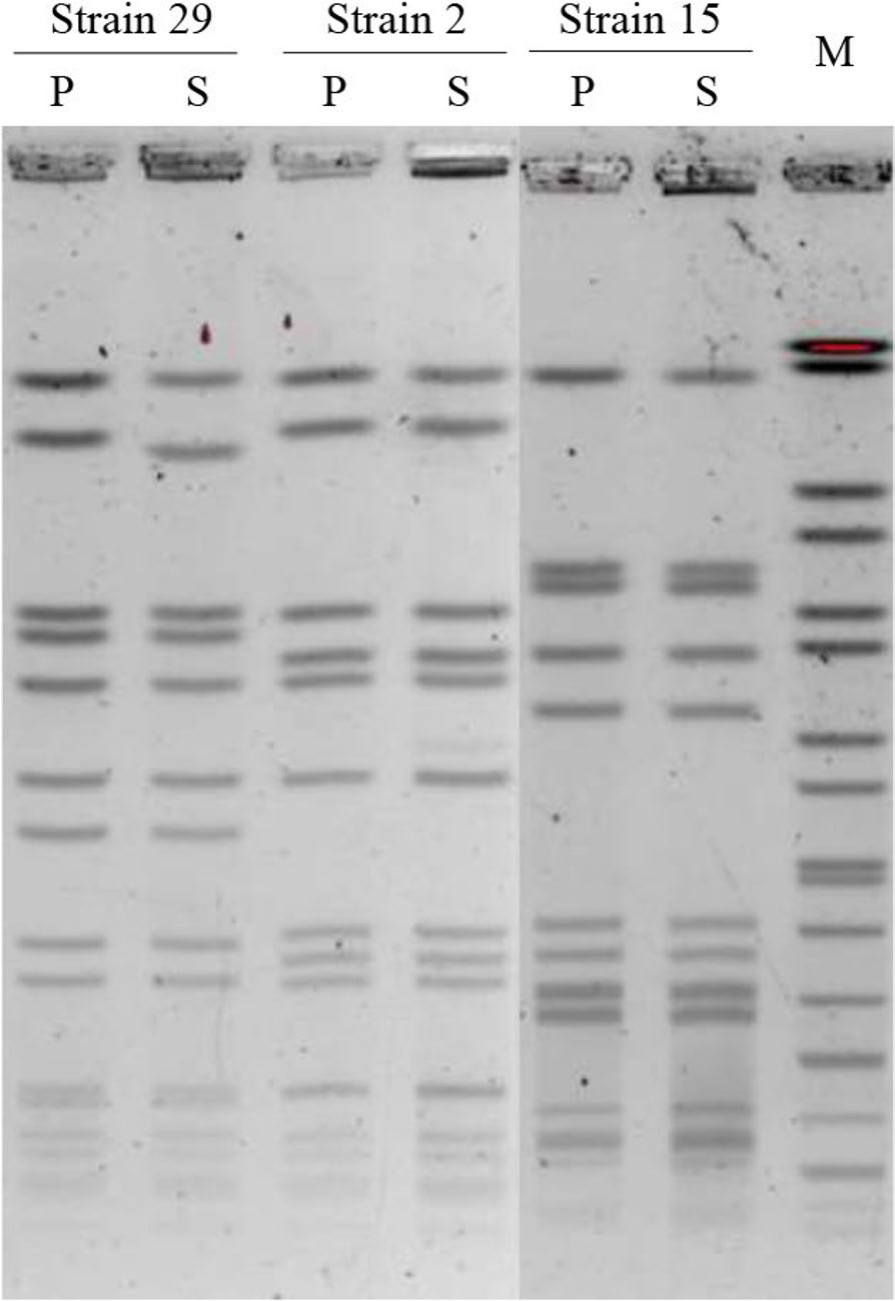
Pulsed-field gel electrophoresis (PFGE) patterns (SmaI digest) of three pairs of *S*. *aureus* isolates. P represents the parent normal *S*. *aureus*, S represents the small-colony variant (SCV), and M represents the molecular marker

The 3 selected SCVs were confirmed to be *S. aureus* through Vitek MS detection and 16S rRNA sequencing. PFGE analysis revealed that the induced SCVs exhibited the same pattern profiles as their parental strains (Fig. 2), indicating clonality between the SCVs and the wild-type strains [11]. Additionally, *agr* typing showed that the induced SCV and its parental strain belonged to the same type (strains 2 and 29: *agr* type II; strain 15: *agr* type I). This information confirmed that the SCVs originated from their respective parental strains.

### Thymidine, menadione, and hemin compensation in induced SCVs

Since the formation of *S*. *aureus* SCVs are usually linked with deficiencies in the utilization of menadione, hemin or thymidine by bacteria, we conducted the auxotrophism test on the induced SCVs to find the possible mechanisms [12]. The results showed that the colony sizes of the 3 induced SCVs strains on MH plates supplemented with each compound separately were not significantly different from those on the plates without these agents, as shown in Fig. S1. This indicated that our SCVs were not dependent on thymidine, menadione, or hemin for their growth.

### Growth curves of SCVs

The growth kinetics of SCVs in TSB were examined, as depicted in Fig. S2. Compared to the control parental strains, the SCVs exhibited a noticeably reduced in vitro growth over the monitoring period. Particularly for the SCVs derived from strains 15 and 29, their OD_600_ values at 16 h were only 55.3% and 42.7% of those observed in their respective parental strains.

### Antimicrobial susceptibility

Commonly used antibiotics MICs of the isolates as determined by E-test are shown in Table S3. The results showed that the MICs of penicillin to SCV 29, linezolid to SCV 2, oxacillin, ciprofloxacin, vancomycin and clindamycin to SCV 15, gentamicin and amikacin to SCVs 15 and 29, tigecycline to SCVs 2 and 29, and rifampicin and SXT to three SCVs were elevated when compared with corresponding parental strains. There was no change in the susceptibility of levofloxacin and erythromycin for all SCVs.

### Analysis of genome sequence and single nucleotide polymorphisms

Based on the kinetics curves of bacterial growth and the timing of SCV formation, strain 15 (day 1) and its corresponding SCV (day 15) were chosen for whole-genome sequencing analysis. The genome features of both strains are summarized in Table 1. A phylogenetic tree reveals a close relationship between strain 15 and its SCV form with strains of *S. aureus* USA300 FPR3757, COL, and NCTC 8325 (Fig. S3). Mauve analysis shows a few observed genomic rearrangements in the SCV compared to the parental strain, potentially resulting from genome shuffling (Fig. S4).

**Table 1:** The comparative genomic features between the SCV and its parental *S*. *aureus* strain (strain 15)

**Strain**	**Genome size (bp)**	**Contig**	**Contig N50 (bp)**	**G + C content (%)**	**rRNA**	**tRNA**	**Other RNA**	**gene**	**CDS**
Strain 15	2,847,287	74	108,842	32.6	4	104	48	2821	2665
SCV	2,835,490	63	153,523	32.6	4	99	52	2810	2655

The sequencing results revealed that the genome of the parental strain was assembled into a single contig measuring 2,847,287 base pairs in size, with an average GC content of 32.62% and containing 2,665 coding DNA sequences (CDSs). On the other hand, the SCV genome had a size of 2,835,490 base pairs, an average GC content of 32.62%, and 2,655 CDSs. Contig N50 refers to the length of the shortest contig that, when combined with all contigs of equal or greater length, covers 50% of the entire genome assembly. The abbreviations rRNA and tRNA stand for ribosomal RNA and transfer RNA, respectively. Finally, CDS represents coding DNA sequence

Identification of SNPs, insertions, and deletions revealed 29 mutation events in the SCV compared to its parental strain, with half of them being synonymous mutations. These missense mutations primarily affected 13 genes, including *spa*, *clfB*, *deoB*, *mgtE*, *asp2*, and those located on phage genome (*holin*, SAOUHSC_01556, 02022, 02025, 02029, 02074, 02076, and 02216). The mutation events are listed in Table 2.

**Table 2:** Single nucleotide polymorphisms (SNPs) in SCV compared with its parental strain (strain15) by whole-genome sequencing

Locus ID	Gene symbol	Nucleotide change	Amino acid change	Product	Pathway
SAOUHSC_00069	*spa*	1063_1064delGGinsAA	Gly355Asn	Protein A	*S. aureus* infection
SAOUHSC_00101	*deoB*	C295T	Stop_gained, Gln99*	Phosphopentomutase	Pentose phosphate pathway; Purine metabolism; Metabolic pathways
SAOUHSC_00398	*hsdS*	A33G	No change	Restriction modification system specificity subunit	
SAOUHSC_00544	*sdrC*	C2433T, T2442G, C2445T, 2283_2289delTTCGGATinsCTCAGAC	No change No change	Fibrinogen-binding protein SdrC	*S. aureus* infection
SAOUHSC_00545	*sdrD*	A3564G, T3567C	No change	Fibrinogen-binding protein SdrD	*S. aureus* infection
SAOUHSC_00945	*mgtE*	C315G	Asp105Glu	Magnesium transporter	
SAOUHSC_02963	*clfB*	A2274G, C2262T, G2253A, A2256G A2319C C1899G	No change Glu773Asp Asp633Glu	Clumping factor B	*S. aureus* infection
SAOUHSC_02987	*asp2*	A188C	Gln63Pro	Accessory Sec system protein Asp2	
SAOUHSC_01552		G126A	No change	Bacteriophage L54a deoxyuridine 5-triphosphate nucleotidohydrolase	Pyrimidine metabolism; Metabolic pathways; Nucleotide metabolism
SAOUHSC_01553		A96G, T324G	No change	PVL orf 52-like protein	
SAOUHSC_01556		A270C	Lys90Asn	PVL orf 52-like protein	
SAOUHSC_02020		G7A	Val3Ile	Holin	
SAOUHSC_02022		1107_1110delCAGTinsAACA A964T A961G	Ser370Thr Thr322Ser No change	Phage tail fiber protein	
SAOUHSC_02025		216_219delTATGinsCATA	Met73Ile	Phi SLT orf 99-like protein	
SAOUHSC_02029		435_436delGCinsAA C150T	Gln146Lys No change	Phi ETA orf 56-like protein	
SAOUHSC_02033		A3042T	No change	Phage tape measure protein	
SAOUHSC_02064		C138T	No change	Phi ETA orf 25-like protein	
SAOUHSC_02074		256_258delGATinsAAC	Asp86Asn	Phi PVL orf 39-like protein	
SAOUHSC_02076		G146A A141G	Cys49Tyr No change	Phi PVL orf 38-like protein	
SAOUHSC_02086		A165T	No change	PV83 orf 4-like protein	
SAOUHSC_02211		A357G	No change	Phi PVL orf 50-like protein	
SAOUHSC_02216		4_6delATTinsGTA	Ile2Val	Phage DnaC-like protein	

### Identification and DEGs

**Fig. 3:**
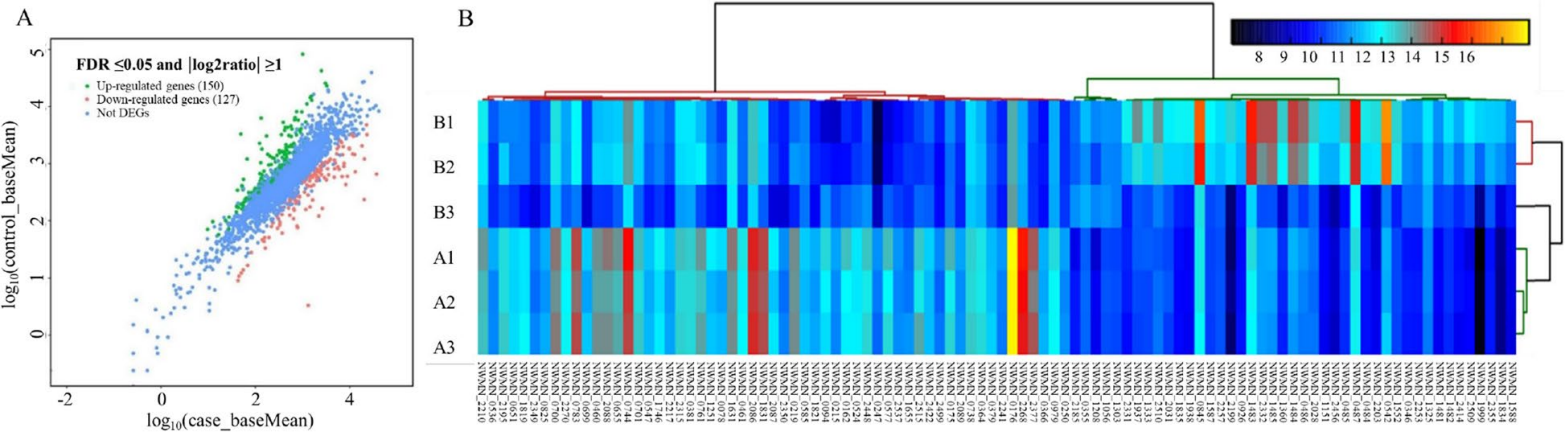
RNA Sequencing analysis of DEGs between parental *S*. *aureus* strain 15 and its SCV. **A** Scatter plot illustrating the RNA sequencing analysis of DEGs between the parental *S*. *aureus* strain 15 and its induced SCV. The DESeq2 v 1.10.1 package was employed to identify the DEGs, considering a |log2(fold change)|≥ 1 and a false discovery rate (FDR) ≤ 0.05. The x- and y-axes represent the normalized signal values of samples in the two groups. Genes up-regulated by the SCV are denoted by green dots, while genes down-regulated by the SCV are represented by red dots. Blue dots indicate genes that were not differentially expressed. **B** Heat map displaying the DEGs between the SCV and its parental strain. A1-A3 correspond to parental strain samples, while B1-B3 represent SCV samples. DEGs stands for differentially expressed genes. Each box in the heat map represents a gene, with the color indicating the level of gene expression. Each column represents the expression of each gene in different samples, and each row represents the expression of all genes in each sample

A total of 54,727,711 raw reads were obtained through RNA sequencing. After quality checking, 39,294,597 reads (19,342,594 for the parental group and 19,952,003 for the SCV group) were generated (Table 3). Subsequent analysis of gene expression profiles identified 2,418 expressed genes, among which 277 (11.5%) were differentially regulated, including 150 significantly increased and 127 prominently decreased DEGs (Table S4). Figure 3 displays a visualization of all the DEGs between the SCV and its parental strain. The heat map (Fig. 3B) clearly demonstrates several genes that were markedly down-regulated in the SCV, such as NWMN_2268 (encoding lactate permease) and NWMN_0176 (encoding L-lactate dehydrogenase), both of which play crucial roles in metabolic pathways. The representative DEGs are listed in Table S5.

**Table 3:** The data of RNA sequencing after quality control for samples of *S. aureus* strain and its SCV

**Sample**	**R1_reads**	**R1_bases**	**R2_reads**	**R2_bases**	**Q20 (%)**	**Q30 (%)**
Strain 15 A1	6,319,209	938,642,415	6,319,209	934,798,560	98.87	96.41
Strain 15 A2	6,365,394	945,609,322	6,365,394	942,566,596	98.93	96.58
Strain 15 A3	6,657,991	989,464,204	6,657,991	984,168,431	98.87	96.40
SCV B1	8,350,568	1,239,642,752	8,350,568	1,232,397,416	98.79	96.06
SCV B2	7,043,490	1,045,256,354	7,043,490	1,040,832,691	98.82	96.18
SCV B3	4,557,945	676,162,167	4,557,945	673,879,525	98.87	96.41

The RNA sequencing data for clinical *S*. *aureus* strain 15 and its SCV included two sequences for each sample, corresponding to the forward (R1) and reverse (R2) end sequences. The table presented displays the data after the bases underwent quality checking using Trimmomatic. The sequencing quality values provided, Q20 (%) and Q30 (%), indicate that the error rate of the bases was less than 1% and 0.1% respectively. The sample numbers are denoted as An and Bn

**Fig. 4:**
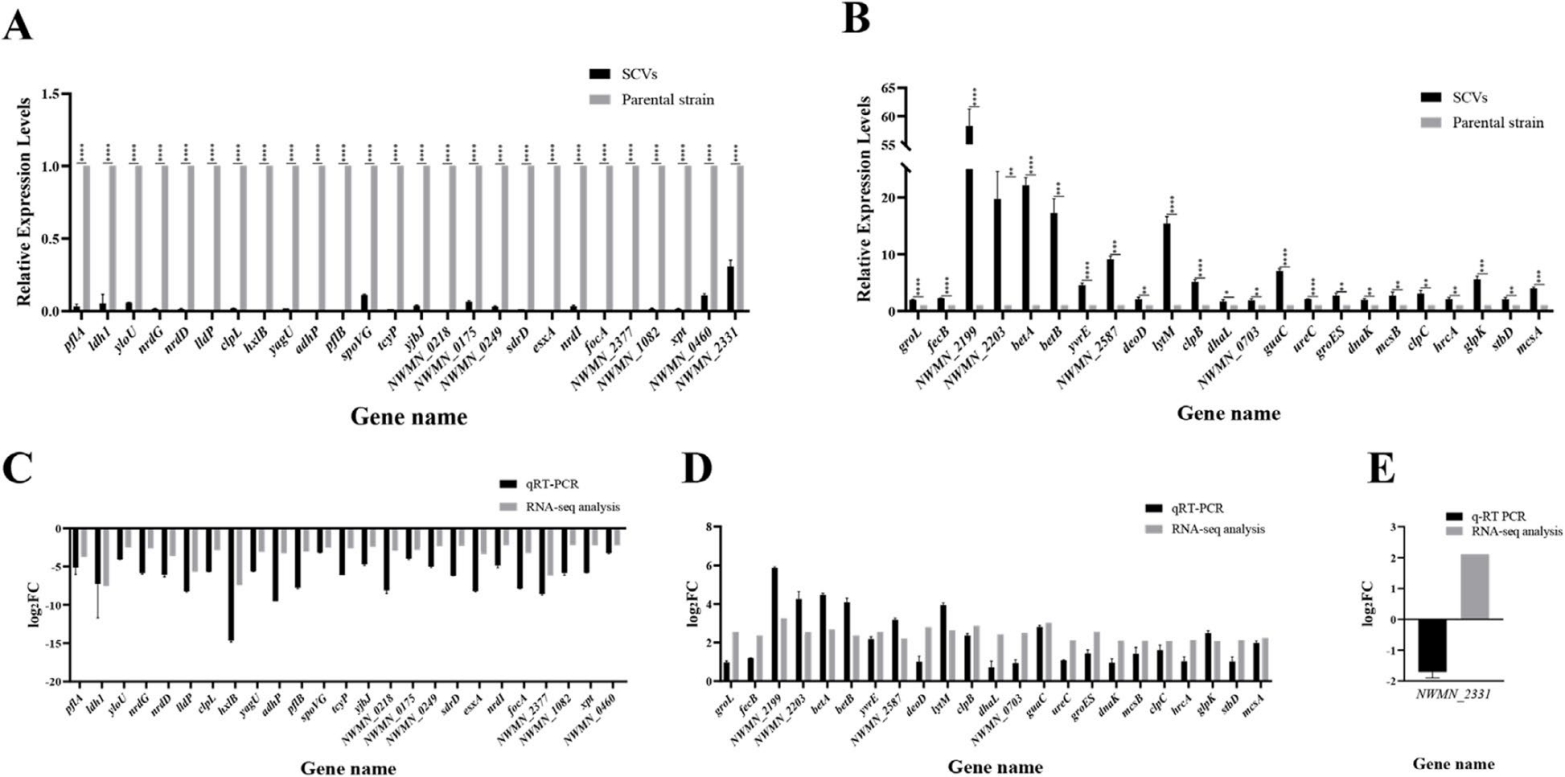
qRT-PCR analysis for confirmation of transcription levels of selected 48 DEGs between SCV and its parental strain. **A** qRT-PCR analysis revealed that 23 DEGs were up-regulated in the SCV. **B** 25 DEGs were found to be down-regulated in the SCV. The black bar represents SCVs, while the gray bar represents the parental strain. Significance levels: *, *P* < 0.05, **, *P* < 0.01, ***, *P* < 0.001, ****, *P* < 0.0001. The conformance of the data between RNA sequencing and qRT-PCR, analyzed with log2FC (y-axis), is depicted in panels C, D, and E. **C**, **D** The verification of 23 up-regulated and 25 down-regulated determinants in the SCV showed matching results between the two detection technologies. **E** The expression of NWMN_2331, as assayed by qRT-PCR, was confirmed to be opposite to that observed in RNA sequencing. The black bar represents qRT-PCR, while the gray bar represents RNA sequencing. qRT-PCR stands for quantitative real-time polymerase chain reaction, while DEGs refers to differentially expressed genes. FC represents fold change

To validate the transcriptome results, the expression levels of 49 selected genes (23 up-regulated and 26 down-regulated genes) were reanalyzed using qRT-PCR. As shown in Fig. 4, the transcription levels of 48 out of 49 DRGs (98.0%) validated by qRT-PCR were consistent with the findings from RNA sequencing. Contradictory data (RNA sequencing: up-regulated; qRT-PCR: down-regulated) were observed for the gene NWMN_2331 (Fig. 4E). Although there were isolated discrepancies between the two technologies, overall, the transcriptome data were reliable.

### Bioinformatics analysis

#### GO enrichment analysis of DEGs

**Fig. 5:**
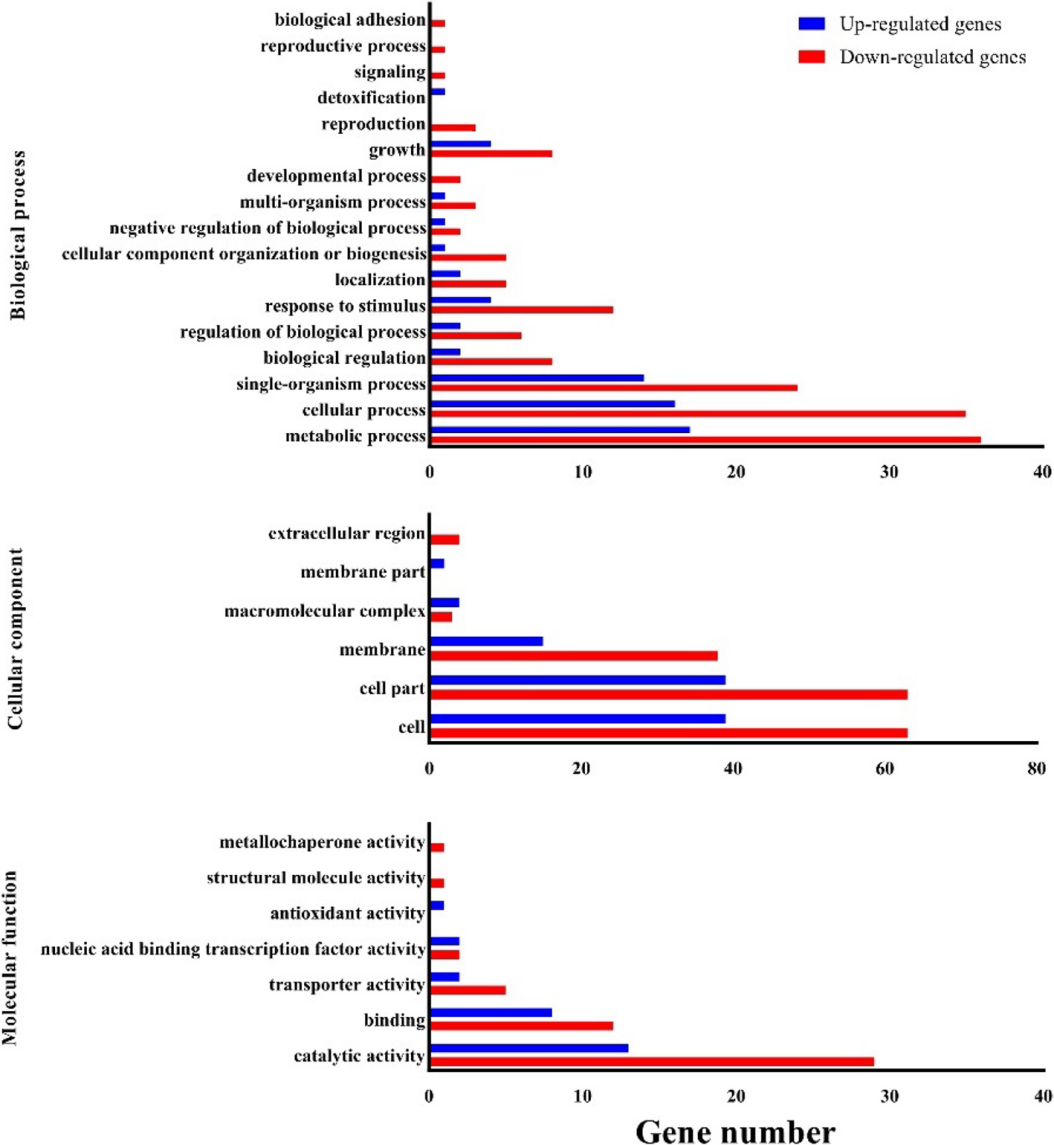
GO enrichment analysis of DEGs between SCV and its parental strain. GO databases were utilized to perform GO functional annotation on the DEGs using the R package GOseq v 1.18. The annotated determinants (y-axis) are categorized into three GO domains: biological process (17 terms), cellular component (6 terms), and molecular function (7 terms). The x-axis represents the number of DEGs. The blue histogram corresponds to the up-regulated genes, while the red histogram represents the down-regulated genes. GO stands for Gene Ontology

The GO enrichment analysis revealed that the DEGs were enriched in three GO domains: biological process (63 terms), cellular component (109 terms), and molecular function (55 terms) (Table S6). Further exploration showed significant enrichment in certain GO items. For example, the biological process domain had a notable enrichment of genes involved in metabolic process (53 genes), cellular process (51 genes), and single-organism process (38 genes). In the cellular component domain, enrichment was observed in cell and cell part (102 genes each), as well as membrane (53 genes). The molecular function domain showed enrichment in catalytic activity (42 genes) and binding (20 genes) (Fig. 5, Table S6). Most of the GO items for biological functions were enriched with down-regulated genes in SCV, and there were almost twice as many the down-regulated genes as the up-regulated genes (Table S6). Besides, from GO analysis, we could find that down-regulated genes were significantly enriched in “response to stimulus”, “growth” and “membrane part” categories, which may imply that dysfunction in these functions occurred in SCV.

#### KEGG pathway enrichment analysis

**Fig. 6:**
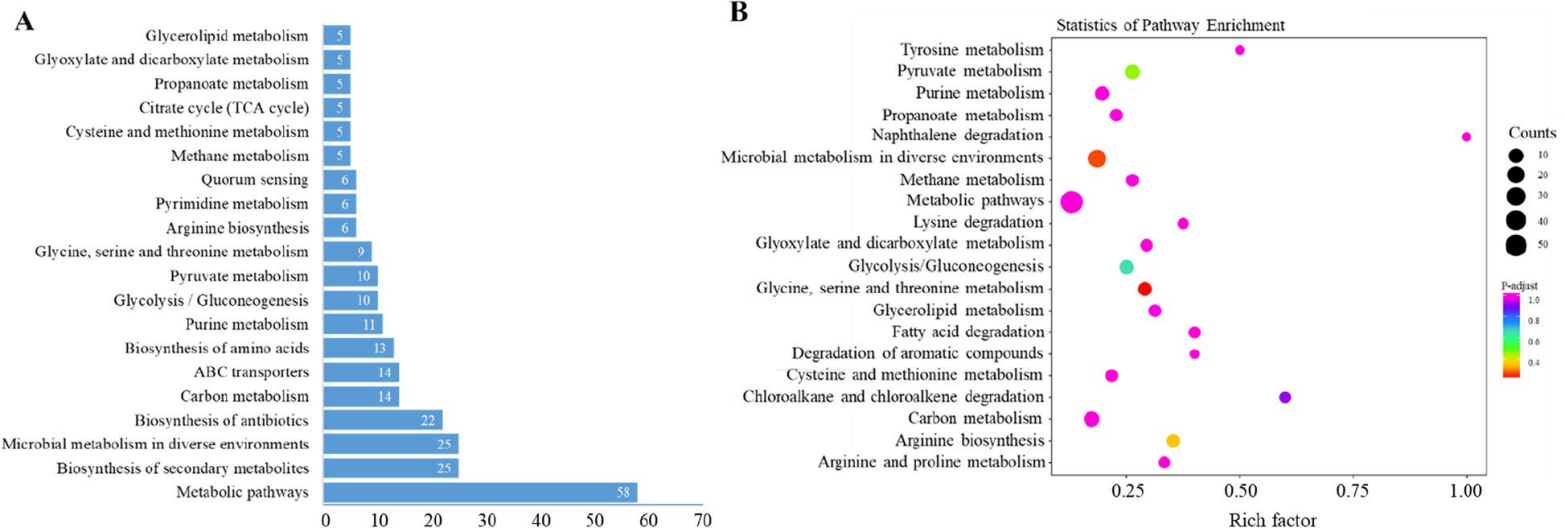
Enrichment analysis of KEGG pathways for DEGs between SCV and its parental strain. The enrichment analysis of KEGG pathways was conducted using the KEGG database. **A** The top 20 enriched KEGG pathways of the DEGs are presented. These pathways are arranged in order of the number of DEGs, with metabolic pathways being the most enriched, followed by biosynthesis of secondary metabolites, microbial metabolism in diverse environments, and biosynthesis of antibiotics. **B** Scatterplots depict the 20 most significant KEGG enrichment pathways of the DEGs. The selection of these pathways was based on three factors: the rich factor corresponding to each pathway (horizontal axis), the p-adjust value (color of the dots), and the number of DEGs enriched in each pathway (size of the dots). The vertical axis represents the names of the pathways. KEGG refers to the Kyoto Encyclopedia of Genes and Genomes

Mapping the DEGs to KEGG pathways identified 68 different pathways associated with 94 DEGs (Table S7). As shown in Fig. 6A, the most prevalent pathways among the DEGs were metabolic pathways (58 genes), followed by biosynthesis of secondary metabolites (25 genes), microbial metabolism in diverse environments (25 genes), and biosynthesis of antibiotics (22 genes). Additionally, pathways related to carbon metabolism (14 genes), ABC transporters (14 genes), biosynthesis of amino acids (13 genes), purine metabolism (11 genes), glycolysis/gluconeogenesis (10 genes), pyruvate metabolism (10 genes), and glycine, serine, and threonine metabolism (9 genes) were significantly enriched (Fig. 6A). Furthermore, a few DEGs were associated with arginine biosynthesis, pyrimidine metabolism, quorum sensing (6 genes each), methane metabolism, cysteine and methionine metabolism, citrate cycle, propanoate metabolism, glyoxylate and dicarboxylate metabolism, and glycerolipid metabolism (5 genes each). The bubble chart in Fig. 6B illustrates 20 significantly enriched pathways based on three dimensions: rich factor, *p*-value, and gene number.

#### Analysis of differential metabolites

**Fig. 7:**
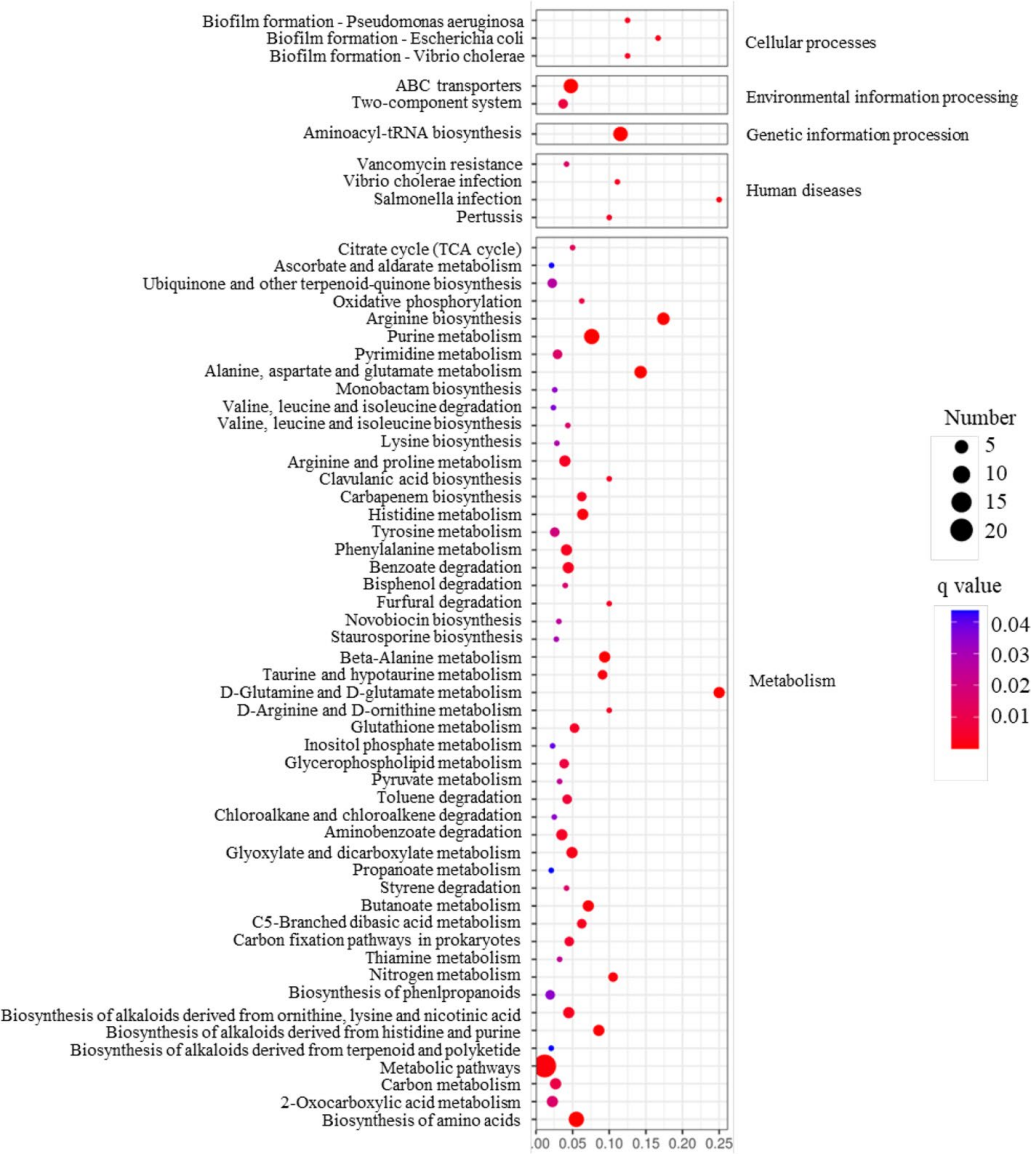
KEGG analysis of differentially regulated ions between SCV and its parental strain. In the bubble chart, the 60 most significantly enriched pathways are displayed out of a total of 73 enriched pathways. The selection of these 60 pathways was based on the number of metabolites, p-value, and enrichment factor. The enrichment factor represents the ratio of differential metabolites located in a specific KEGG pathway to the total number of metabolites in that pathway. A smaller p-value indicates a higher level of KEGG enrichment

After processing the raw mass spectrometry data using XCMS, a package based on the R language, we utilized metaX, a metabolomics data analysis software also based on the R language, along with KEGG to obtain the first-level identification results of metabolites. Out of the 27,176 identified metabolites, 2,992 differential ions (11%) were characterized, including 1,345 up-regulated ions (4.9%) and 1,647 down-regulated ions (6.1%) in the SCV (Table S8). It is worth noting that the ions with the highest fold of up-regulation in the SCV were all associated with carboxylic acids and derivatives **(**Table S9). The KEGG pathway analysis revealed that the differentially regulated ions were primarily enriched in pathways related to metabolism, with only a small fraction associated with cellular processes, environmental information processing, genetic information processing, and human disease (Fig. 7). Among the metabolic pathways, the three most enriched pathways in terms of ions were metabolic pathways (34 differentially regulated ions), biosynthesis of secondary metabolites (16 differentially regulated ions), and biosynthesis of amino acids (13 differentially regulated ions). Additionally, ABC transporters from environmental information processing and aminoacyl-tRNA biosynthesis from genetic information processing each enriched 11 differentially regulated ions.

#### Analysis of transcriptomic and metabolomic data

**Fig. 8:**
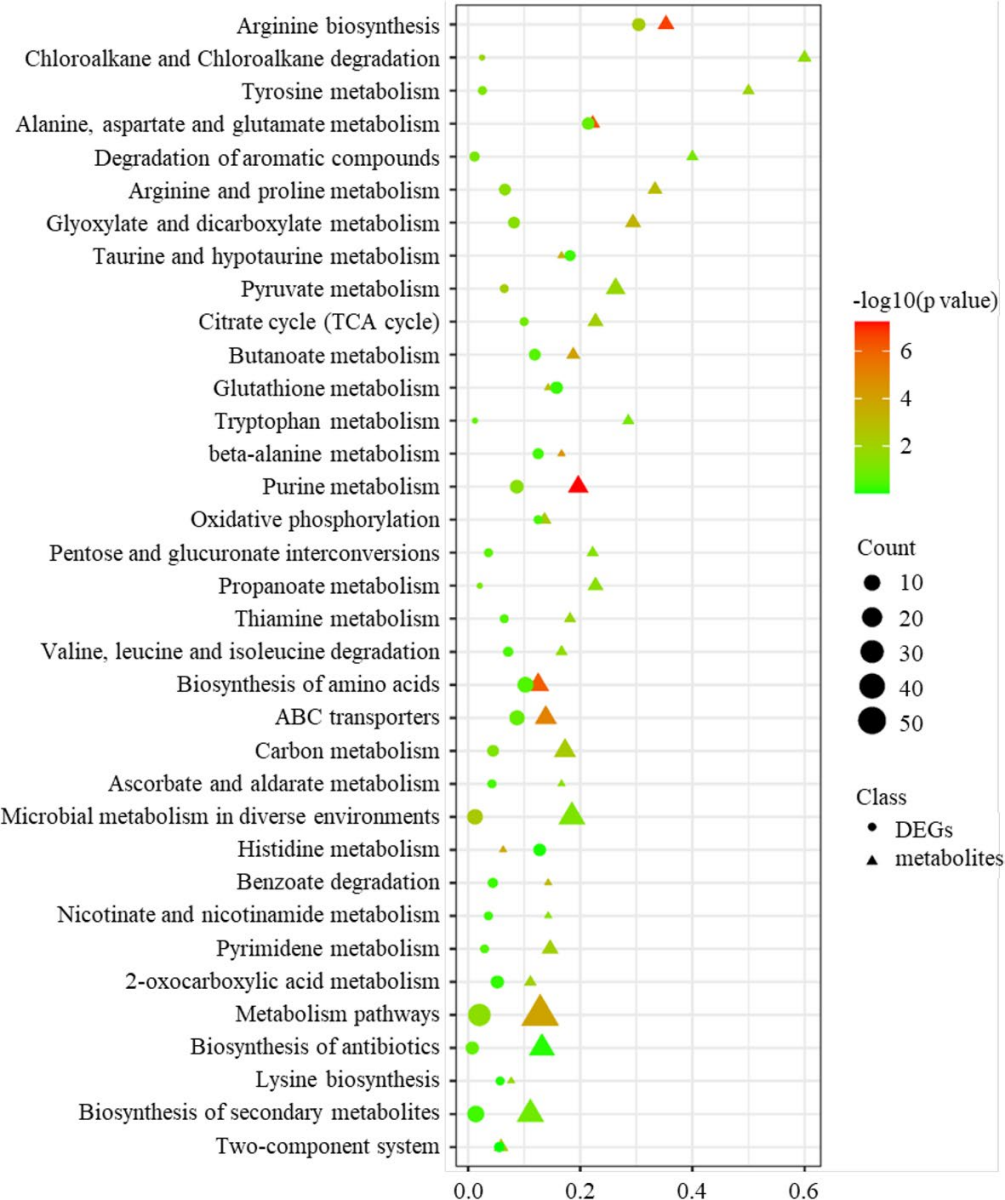
Bubble chart of overlapped pathways in KEGG analysis. This figure displays a bubble chart representing the 35 pathways that overlap between the metabolomic and transcriptomic data in the KEGG analysis. In the chart, DEGs are marked with circles, while differentially regulated metabolites are marked with triangles. The enrichment factor is a measure indicating the ratio of differential genes/metabolites located within a specific KEGG pathway to the total number of genes/metabolites in that pathway. A smaller p-value corresponds to a higher level of KEGG enrichment

The conjoint analysis of transcriptomic and metabolomic data utilized DEGs and significantly different metabolites (SDMs) that were enriched in KEGG pathways. A heatmap, generated using the Spearman method, was used to visualize the correlation between DEGs and SDMs (Fig. S5). By examining the overlapping pathways, a total of thirty-five pathways were identified, with the majority being involved in metabolic pathways (Fig. 8). Notably, the highlighted common pathways included purine metabolism, pyruvate metabolism, ABC transporters, and arginine metabolism. These pathways had an impact on bacteria in terms of nucleic acid synthesis and energy metabolism. For instance, out of the 14 DEGs from ABC transporters, 10 were connected to the iron complex transport system permease protein. Furthermore, 11 SDMs, which included several amino acids, are important components of ABC transporters.

## Discussion

Although constructing a mutant of the action site of SXT can result in a stable phenotype of SCVs, it is challenging to accurately reflect the actual clinical situation [13, 14]. In this study, we induced clinical *S. aureus* isolates with SXT to generate stable SCVs by mimicking clinical anti-infective treatment, and explored the possible formation mechanisms of SCVs by phenotype and omics analysis. Genes mutation involved in the biosynthesis of menadione (*menB*-*F*, *aroD* and *ispA*), heme (*hemA*, *B*, *E*, *G*, *H*, *Q*, *hrtB*, *A*, *cydB*, *qoxB*, and *ctaA*), and thymine (*thyA*) are known to play a critical role in SCV formation [7, 15]. In recent years, mutations in genes related to fatty acid metabolism (*acpP*, *fabF*, *accD*, *plsX*, *fakA*, and *fakB*), tricarboxylic acid cycle (*ndh2, sdhCAB, mpsA, cydAB*), oxidative stress (*mpsB*, *mpsABC*, *rubU*, and *mgrA*), DNA proofreading and repair (*mutS*), regulation (*agr*, s*igB*, *sarA* and *codY*), and rifampicin resistance (*rpoB*) have also been reported to be associated with the *S. aureus* SCVs development [15, 16]. In this study, the auxotrophism test showed no connection between the auxotrophism of menadione, hemin and thymine and the induced SCVs. Subsequent genome sequencing results of representative SCV also confirmed the absence of mutations in the genes involved in the aforementioned biopathways, and physiological and biochemical processes [15]. However, a few missense mutations were observed in genes correlated to virulence (such as *spa*, *clfB*, and phage-encoded factors), nucleotide metabolism (*deoB*), and transport proteins (*asp2* and *mgtE*). Surfactant protein A (encoded by *spa*) and clumping factor B (encoded by *clfB*) contribute to the adhesion, colonization and host immune evasion of *S*. *aureus* [17, 18]. Kriegeskorte et la. [13] reported that the expression of both genes was up-regulated in a thymidine-dependent SCV caused by *thyA* inactivation. Therefore, the missense mutations in *spa* and *clfB* might indicate a possible alteration in the pathogenicity of our SCV.

Bacterial virulence factors encoded by phages or phage-related regions have been described in *S. aureus* [19], which can be lethally toxic (such as HolGH15) or modify the bacterial status [20, 21]. The loss of phage in the genome of *S*. *aureus* has been verified to be related to SCV formation [16]. Considering these findings, it is difficult not to speculate that the missense mutations in several phage-related regions, which encode toxins and phage structural proteins, may contribute to the formation of SXT-induced SCV in this study.

Another noteworthy observation is the presence of non-synonymous mutations in *mgtE* (C315G, converting at the protein-level to Asp105Glu) and *deoB* (C295T, and Gln99*, * stands for a stop codon) in the SCV. Xu et al. [22] have indicated that silencing of *mgtE* leads to a decrease in cytoplasmic levels of Mg^2+^ and ATP, thereby further inhibiting the growth of *S. aureus*. DeoB, namely phosphopentomutase, plays a role in catalyzing the intramolecular transfer of phosphate on ribose or deoxyribose, and is involved in the salvage pathway of nucleoside biosynthesis [23]. Therefore, mutations in these two genes could potentially lead to their functional defects, and further promote the development of SCVs [15, 23, 24]. The speculation about the role of *deoB* mutation can be supported by Chen's study [25], who found mutations in *deoC*, another gene in the pentose phosphate pathway, in a vancomycin induced SCV.

Our transcriptomics data indicated that the majority of the DEGs are involved in metabolic pathways. Of particular interest is the KEGG pathway of ATP binding cassette (ABC) transporters, which is enriched with 14 DEGs. These include 5 genes encoding iron complex transport system (ICTS) permease protein (K02015) such as FhuB, 3 genes encoding ICTS substrate-binding protein (K02016) such as FhuD, and 2 genes encoding ICTS ATP-binding protein (K02013) such as FhuC. FhuC works in conjunction with FhuB, FhuG, and FhuD to transport iron across the cell membrane, and its mutation has been found to reduce the growth and the virulence of *S. aureus* [26]. Notably, the expression of all the DEGs involved in ABC transporters aforementioned was down-regulated in our SCV. Additionally, the metabolomics data revealed the enrichment of 11 differential metabolites in ABC transporters, predominantly amino acids such as arginine, L-glutamine, and histidine. These findings suggested that changes in ABC transporters may be one of the main factors contributing to the formation of SCV. The similar significance related to ABC transporter family was also reported in the vancomycin induced SCV [27]. ABC transporter proteins belong to the pump protein family, and are closely associated with the biofilm formation and resistance of *S. aureus* [28, 29]. A previous study demonstrated a reduction in virulence in strains with dysfunction of the Ecs ABC transporter [30]. Therefore, the changes in the expression of genes encoding these proteins may also affect the virulence and resistance characteristics of our SCV.

*S. aureus* relies on the glycolysis/gluconeogenesis pathway to generate ATP for its life support requirements [31]. It is reported that the glycolysis/gluconeogenesis pathway differs between SCVs and their prototype strains [32]. Our data show that 10 DEGs are significantly enriched in this pathway. Of particular interest, the SCV exhibited a remarkable decrease by180-fold in the expression of *ldh* encoding lactate dehydrogenase. LDH is a crucial enzyme involved in the fermentation of pyruvate to lactate, and appears to be a major factor contributing to *S. aureus* growth under nitrite stress [33]. Therefore, the impaired growth of SCVs may be linked to the significant reduced expression of *ldh*.

Previous research has demonstrated that MgrA, a global regulator, positively regulates the expression of exoproteins and negatively modulates the expression of surface proteins [34]. Bui et al. [35] analyzed the role of MgrA, and discovered a SNP (G to A, and Arg94Cys) in its encoding gene, which might render the gene inactive and contribute to the formation of the stable SCV induced by chemical stresses. Although no mutation of *mgrA* was observed in this study, the down-regulated transcriptional level of it in the SCV suggests the potential significance of MgrA in the development of our SCV.

In addition to the intrinsic genetic alterations explored through genomics and transcriptomics, metabolomics analysis revealed significant metabolic differences between the SCV and its parental strain, particularly in ABC transporters, aminoacyl-tRNA biosynthesis, and purine metabolism. Further integration analysis of the transcriptomic and metabolomic data confirmed the findings of both omics. Among these pathways, purine metabolism stood out due to its profound impact on *S. aureus* growth [36]. In the transcriptomic data, the expression of genes involved in the de novo biosynthesis of guanine nucleotides, such as *guaA, guaC, xpt,* and *deoD*, was found to be altered (Table S4). The metabolomic results showed enrichment of 8 ions related to purine metabolism, including L-glutamine, adenine, adenosine, guanine, hypoxanthine, 3',5'- and 2',3'-cyclic AMP. These suggest that variations in purine metabolism, particularly guanine metabolism, may be one of the core mechanisms in the formation of SXT-induced SCV tested. Previous research has reported that arginine catabolism can facilitate *S. aureus* growth in the host [37]. In this study, we observed an increase in the amount of arginine and the deregulation of arginine-related gene expression in the SCV. These findings indicate multifaceted changes between the SCV and its parental strain studied, including disturbances in nucleotide synthesis and energy metabolism.

It was reported that slower bacterial growth exhibit increased resistance to antimicrobials [7, 38, 39]. Previous data showed that the MICs of aminoglycosides for SCVs are 8 to 32 folds higher than those of their parental strains [38]. However, the MICs of gentamicin and amikacin for the SCVs 15 and 29 were only elevated 2 to 4 times. Fluoroquinolones generally have little effect on the change of MICs of SCVs [39], and the MICs of ciprofloxacin and levofloxacin of SCV 15 (0.125 μg/ml and 0.5 μg/ml, respectively) and its parental strain (0.064 μg/ml and 0.5 μg/ml, respectively) in this study were consistent with this view (only 0 to twofold changes). For other drugs, we observed that the MIC of penicillin to SCV 29, the MIC of linezolid to SCV 2, the MICs of oxacillin, vancomycin and clindamycin to SCV 15, the MICs of tigecycline to SCV 2 and 29, the MICs of rifampicin and SXT to SCV 2, 15 and 29 were increased by 2 (vancomycin) to 4000 (rifampicin) -fold compared with their parental strains. The reasons why our SCVs have a great increase in the MICs of some antibacterial drugs is unknown, and the mechanisms is worthy of in-depth exploraton.

## Conclusion

The switching and development of *S. aureus* to SCV phenotype is significant to its pathogenesis and persistent ​survival in the host. This study described the phenotype features and multi-omics landscape of the *S. aureus* SCVs produced through SXT screening. Classical determinants associated with the formation of *S. aureus* SCVs did not play a role. However, we did observe a few non-synonymous variants in genes involved in adhesion, nucleoside biosynthesis, Mg^2+^ and ATP metabolism, and phage components, etc., whose role in SCV formation needs further investigation. By analyzing the DEGs and the SDMs, we identified metabolic pathways like purine metabolism, pyruvate metabolism, ABC transporters, and arginine metabolism that potentially affect nucleic acid synthesis and energy metabolism in *S. aureus*, thus influencing SCV formation. Additionally, we also observed that SCV strains exhibited stronger resistance than their corresponding parental cell-types. It should be noted, however, that there is a limitation to this study. We did not conduct multi-omics analysis on all SCVs and their parental strains, which may lead to a bias in the comprehensive understanding of the relevant changes involved in the development of SCV. Despite this limitation, our results can still provide some new insights into the internal changes of SCV in *S. aureus* under SXT pressure, laying a foundation for a more in-depth investigation of the mechanisms behind the phenotypic switch from wild-type to SCV. In addition, in clinic a variety of antimicrobial agents can induce *S*. *aureus* to switch to SCV, and the switching mechanism induced by different drugs may be different [40]. Therefore, the results in this study may not necessarily reflect the situation of other drugs.

## Methods

### Bacterial strains

A total of 30 non-replicate clinical isolates of *S. aureus* were randomly selected from blood and pus samples of different inpatients for the induction of SCVs phenotype. Table S1 provides the relevant information for each of the isolates. As the focus of this study is on microorganisms, it was exempted from review by the Ethics Committee of Shanghai General Hospital.

### Preparation of trimethoprim-sulfamethoxazole

Trimethoprim (TMP) (Sigma, St. Louis, USA) was dissolved in a small amount of 10% lactic acid and then added to sterile distilled water to create a storage solution with a concentration of 1200 μg/ml. Sulfamethoxazole (SMZ) powder (Sigma) was covered with sterile water. A small amount of 5 mol/L NaOH solution was then added, and the mixture was shaken to completely dissolve the drug. Finally, an appropriate amount of sterile distilled water was added to prepare a storage solution with a concentration of 22,800 μg/ml. A working solution of trimethoprim-sulfamethoxazole (SXT) with a final concentration of 12/238 μg/ml was prepared by mixing TMP and SMZ in brain heart infusion (BHI) broth (Oxoid, Hampshire, UK) at a ratio of 1:19 [3].

### Induction of SCVs

A single colony of *S. aureus* was cultured in BHI broth containing 12/238 μg/ml of SXT at 150 rpm/min and 37 °C. After each overnight culture, appropriate dilutions were subcultured into fresh drug-containing BHI broth and incubated with shaking. At the same time, the inoculated cultures were streaked onto drug-free blood plates (Shanghai Kemajia Microbial Technology Co., LTD, Shanghai, China) to observe the bacterial colonies size and growth. The induction and screening experiments were conducted continuously for several days until *S. aureus* with a stable SCVs phenotype was obtained [3]. The stable SCVs phenotype was defined if the small colony morphology persisted over time (after 10 passages) [41].

### Species identification of SCVs

#### MALDI-TOF MS analysis

The species identification of SCVs was performed using matrix-assisted laser desorption/ionization time-of-flight mass spectrometry (MALDI-TOF MS) analysis with the Vitek MS system (bioMérieux, Marcy l’Etoile, France), following the provided instructions. *Escherichia coli* ATCC8739 was used for instrument calibration. All SCVs isolates were identified at the species level by comparing them to the v3.1 database, with confidence values of > 90% considered reliable.

#### 16S rRNA gene sequencing

Fresh colonies of SCVs were ground in 100 μl of TE buffer (10 mM Tris–HCl, 0.1 mM EDTA, pH 8.0) with 5 μl of lysostaphin (1 mg/ml, Sangon Biotech, Shanghai, China). The mixtures were incubated at 37 °C for 35 min, followed by heating at 100 °C for 15 min, and then centrifuged at 12,000 rpm for 10 min. The supernatant containing soluble DNA was used for amplifying the 16S rRNA gene using universal primers 16 s-F (AGAGTTTGATCCTGGCTCAG) and 16 s-R (GGTTACCTTGTTACGACTT) [42]. All PCR products were sent to Shanghai Maple Sequencing Co. for bi-directional sequencing, and the results were compared to the BLAST/NCBI website (https://blast.ncbi.nlm.nih.gov/Blast.cgi) to confirm their specificity to *S. aureus*.

#### PFGE typing

To analyze the clonal relationship between SCVs and their parental strains, the total bacterial DNA was digested with SmaI (Thermo Fisher Scientific, Shanghai, China), and the resulting fragments were resolved using pulsed-field gel electrophoresis (PFGE) with the CHEF Mapper XA system (Bio-Rad, California, USA), following previously described protocols in detail [43]. *Salmonella Braenderup* H9812 digested with XbaI (Thermo Fisher Scientific) was used as the molecular weight marker. The patterns of DNA fingerprints were analyzed using BioNumerics software 7.0.

#### *agr* typing

The internal fragments of the accessory gene regulatory (*agr*) alleles (alleles I to IV) were amplified using the primers listed in Table S2, following previously described methods [44].

#### Auxotrophism test

Auxotrophy was detected by streaking a fresh SCV colony on a Mueller–Hinton agar (MH) plate (Shanghai Kemajia Microbial Technology Co., LTD) supplemented with 1.0 mg/ml of thymidine, menadione, or hemin (Sangon Biotech Co., Ltd., Shanghai, China), based on a modified method described by Yagci [11]. A strain was considered thymidine, menadione, or hemin auxotrophic if growth was observed on the MH plate after 24 h of culture at 35 °C [11].

#### SCV colony growth kinetics

Overnight liquid cultures of SCVs were diluted 1:100 into 25 ml of fresh tryptic soy broth medium (TSB, Oxoid). Cultures of their corresponding parental strains were used as controls. Bacteria were incubated at 37 °C and 150 rpm/min under aerobic conditions. The growth of cultures was monitored by measuring the optical density at 600 nm (OD_600_) every 15 to 30 min using an ultraviolet spectrophotometer (Unico Instruments, Shanghai, China) [45].

#### Antimicrobial susceptibility testing

Antimicrobial susceptibility testing (AST) was conducted using E-test strips (Kont, Wenzhou, China) on MH plates, according to the manufacturer’s recommendations. The minimal inhibitory concentrations (MICs) of SCVs and their parental strains to penicillin, oxacillin, ciprofloxacin, levofloxacin, gentamicin, amikacin, vancomycin, clindamycin, erythromycin, linezolid, rifampicin, SXT and tigecycline were determined. Interpretation of testing results was based on the guidelines of the Clinical and Laboratory Standards Institute (CLSI) [46]. For amikacin and tigecycline, the European Committee on Antimicrobial Susceptibility Testing (EUCAST, v12.0) breakpoint (https://www.eucast.org/) was used. Due to slow growth of SCVs, MICs were also read after 48 h of incubation. *S*. *aureus* ATCC 29213 was used as the control strain.

#### Genome sequencing and bioinformatics analysis

A representative pair of SCV and parental strains were selected for genomic DNA sequencing analysis. Briefly, bacterial genomic DNA was extracted from fresh bacterial cultures using the Quick-DNA Fungal/Bacterial Kits (Zymo Research Corp., California, USA). DNA libraries were prepared using the KAPA Hyper Prep Kit for Illumina® platforms (KAPA Biosystems, Roche®, California, USA) following the manufacturer's protocol and sequenced on the Illumina Novoseq 6000 sequencing platform (Illumina, Inc., California, USA). In genome sequencing, the threshold was set to 15. The quality control of the raw sequences was performed using Trimmomatic (version 0.36) [47]. The clean reads were then assembled using Spades (version 3.14.0) [48] with default parameters. The assembled contigs were rearranged (repositioned and reoriented) using Mauve Contig Mover (MCM) (version 2.4.0) [49] to construct pseudomolecular information based on the *S. aureus* reference genome NC_007795.1 (*S. aureus* NCTC 8325). Finally, the assembled genomes were subjected to Prokka (version 1.14.5) [50] for gene prediction and functional annotation, and Snippy (version 4.5.0, https://github.com/tseemann/snippy) was used to identify single nucleotide polymorphisms (SNPs) and small insertions and deletions (INDELs).

#### RNA sequencing and bioinformatics analysis

RNA sequencing can provide the ability to study the expression of different genes at single-nucleotide resolution, which is a considerable improvement over the major whole-genome transcriptomic techniques used in *S*. *aureus* [51]. The broader application of this technology in research can deepen our understanding of many areas of biology. Therefore, we selected RNA sequencing for the analysis of gene expression. Bacterial cells of representative strain pairs mentioned above in the mid-logarithmic growth phase were harvested by centrifugation at 13,000 × g for 10 min at 4 °C. Total RNA was extracted using the MiniBEST Universal RNA Extraction Kit (TaKaRa, Dalian, China) and an optional on-column DNase treatment procedure (TianGen, Beijing, China), following the manufacturer's instructions. Bacterial mRNA was enriched using the MICROBExpress™ kit (Invitrogen, California, USA) and fragmented for cDNA synthesis. The cDNA was synthesized using the Superscript™ Double-Stranded cDNA synthesis kit (Invitrogen, California, USA) with random hexamer primers (5’-d(NNNNNN)-3’ (*N* = G, A, T or C)). After end-repair, dA-tailing, and adapter ligation, the adapter-modified fragments were amplified to generate the cDNA library using the NEBNext® UltraTM II RNA Library Prep Kit (New England Biolabs, Massachusetts, USA). The cDNA sequencing was performed on the Illumina HiSeq 4000 sequencing platform (Illumina, Inc., California, USA) [52]. The raw data were aligned to the reference genome sequence of NC_009641.1. We utilized the DESeq2 v 1.10.1 package to detect differentially expressed genes (DEGs) between the SCV and its parental strain, with a false discovery rate (FDR) of ≤ 0.05 and |log2(fold change)| of ≥ 1. DEGs were annotated and enriched using the Kyoto Encyclopedia of Genes and Genomes (KEGG) and Gene Ontology (GO) databases (R package GOseq v 1.18) [52]. Data from three biological replicates were used to analyse the difference of gene expression.

#### Validation of DEGs by qRT-PCR

To confirm the RNA sequencing data, a specific number of DEGs were selected for quantitative real-time polymerase chain reaction (qRT-PCR) using primers designed with reference to Genome NC_009641.1 (Table S2). Total RNA was isolated using the TaKaRa MiniBEST Universal RNA Extraction Kit (Takara, Beijing, China) and converted to cDNA using the PrimeScript™ RT reagent Kit with gDNA Eraser (Perfect Real Time) (Takara), following the manufacturer's protocols. The qRT-PCR was conducted on the ABI 7500 Real-Time PCR System (Applied Biosystems, California, USA) using the SYBR Premix Ex Taq™ (Tli RNaseH Plus) kit (TaKaRa), as described by the manufacturer. The housekeeping gene 16S rRNA was used as the reference gene for normalization. Three biological experiments were performed, and the relative gene expression was calculated using the Eq. 2^−ΔΔCT^ [52]. The efficiency of the primers was evaluated through melting curve analysis.

#### Metabolomic analysis

A pair of representative strains mentioned above were cultured at 37 ℃ in TSB medium. After 24 h, the culture supernatants were harvested and stored at -80 ℃. The collected samples were thawed on ice, and metabolites were extracted using a 50% methanol buffer. The metabolomic compositions were analyzed using the Liquid chromatography tandem mass spectrometry (LC–MS/MS) technique (Thermo Fisher Scientific, Waltham, USA) and the corresponding software, following the previously described methods [52]. Six biological experiments of each strain were performed. The biofunctions of the detected metabolites were annotated using the KEGG and human metabolome database (HMDB). The differentially expressed metabolic ions were screened using univariate analysis and variable importance for the projection (VIP) values obtained from partial least squares-discriminant analysis (PLS-DA), a multivariate statistical analysis. The differential ions needed to meet the following conditions: (1) ratio >  = 2 or <  = 1/2, (2) *p*-value ≤ 0.05, and (3) VIP ≥ 1[52].

#### Statistical analysis

We performed statistical analysis using SAS 9.3 software for Windows (SAS Institute Inc., Cary, USA) and R language to analyze the data from all the experiments. Statistical comparisons were carried out using the unpaired Student's t-test.

### Supplementary Information

Additional file 1: Table S1. Information of *S*. *aureus* used for the induction of SCVs. Table S2. Sequences of primers used for PCR in this study. Table S3. The minimal inhibitory concentrations of SCVs compared with those of the corresponding parental strains.Table S4. Transcriptic data. Table S5. The representative DEGs. Table S6. GO data. Table S7. KEGG data. Table S8. All differentially expressed ions in metabolomic analysis. Table S9. KEGG pathhways enriched by differentially expressed ions.

Additional file 2: Figure S1. aureus SCV (strain 15, 2 and 29) on Mueller–Hinton (MH) agar with thymidine, menadione, or hemin. The sizes of the SCV colonies on MH plates with thymidine, menadione, or hemin were similar to those on MH plates without these compounds. This suggests that the SCVs were independent of thymidine, menadione, or hemin. A-D SCV 15. E-H SCV 2. I-L SCV 29. A, E, I Control without any compounds. B, F, J Thymidine. C, G, K Menadione. D, H, L Hemin. Figure S2. Growth curves of three pairs of S. aureus strains and their corresponding SCVs in tryptic soy broth (TSB). The curves clearly demonstrate that the growth of the SCVs was significantly delayed when compared to their corresponding parental strains. Figure S3. Phylogenetic tree depicting the relationship between S. aureus strain 15, its corresponding SCV, and other S. aureus strains. Through phylogenetic analysis, we discovered a strong association between strain 15 and its SCV with S. aureus strains USA300, COL, and NCTC 8325. Figure S4. Mauve alignments showcasing the genomes of S. aureus NCTC 8325, strain 15, and SCV 15. Each colored region represents a locally collinear block (LCB) where no rearrangement of homologous backbone sequences is observed. Mauve analysis revealed a few genomic rearrangement events in the SCV strain compared to the parental strain. Figure S5. Correlation heatmap illustrating the association between DEGs and SDMs enriched in KEGG pathways. The Spearman method was employed for correlation analysis. A higher number of "*" indicates a smaller p-value. The intensity of color and the quantity of "*" within each square reflect the significance of the association between the gene and the metabolite. DEGs, differentially expressed genes; SDMs, significantly different metabolites.

Additional file 3.

## Data Availability

The genome sequences of parental *S. aureus* strain 15 and its SCV can be accessed in the Sequence Read Archive (SRA) under the accession number PRJNA936498. Additionally, the RNA-seq data for parental *S. aureus* strain 15 and its SCV are available in GenBank ( http://www.ncbi.nlm.nih.gov/genbank/) under the accession number PRJNA936833. Researchers interested in accessing these datasets can refer to the provided accession numbers for retrieval.

## Abbreviations

*S. aureus*: *Staphylococcus aureus*
SCV: Small colony variant
SXT: Trimethoprim-sulfamethoxazole
AST: Antimicrobial susceptibility testing
MIC: Minimal inhibitory concentration
PFGE: Pulsed-field gel electrophoresis
*agr*: Accessory gene regulator
GO: Gene ontology
KEGG: Kyoto encyclopedia of genes and genomes
DEG: Differentially expressed gene
SDM: Significantly different metabolites
qRT-PCR: Quantitative real-time polymerase chain reaction
HMDB: Human metabolome database
ICTS: Iron complex transport system
