# Supplementary material for: Integrative omics analysis reveals insights into small colony variants of Staphylococcus aureus induced by sulfamethoxazole-trimethoprim
Source: BMC Microbiol. 2024 Jun 14;24:212. doi: 10.1186/s12866-024-03364-8 (PMC11179224; doi:10.1186/s12866-024-03364-8)
Supplement: Supplementary file 1 — Additional file 1: Table S1. Information of S. aureus used for the induction of SCVs. Table S2. Sequences of primers used for PCR in this study. Table S3. The minimal inhibitory concentrations of SCVs compared with those of the corresponding parental strains.Table S4. Transcriptic data. Table S5. The representative DEGs. Table S6. GO data. Table S7. KEGG data. Table S8. All differentially expressed ions in metabolomic analysis. Table S9. KEGG pathhways enriched by differentially expressed ions. [file 12866_2024_3364_MOESM1_ESM.zip › Table S3 MICs.docx]

**Table S3** The minimal inhibitory concentrations of SCVs compared with those of the corresponding parental strains.

| Strain |  | MICs (μg/ml) | | | | | | | | | | | | |
| --- | --- | --- | --- | --- | --- | --- | --- | --- | --- | --- | --- | --- | --- | --- |
|  |  | PEN | OXA | CIP | LEV | GEN | AMI | VAN | CLI | ERY | LNZ | RIF | SXT | TIG |
| 2 |  | 256^R^ | 256^R^ | 32^R^ | 32^R^ | 256^R^ | 256^R^ | 1^S^ | 32^R^ | 256^R^ | 0.064^S^ | 0.016^S^ | 0.064/1.216^S^ | 0.064^S^ |
| SCV 2 |  | 256^R^ | 256^R^ | 32^R^ | 32^R^ | 256^R^ | 256^R^ | 1^S^ | 32^R^ | 256^R^ | 256^R^ | 32^R^ | 32/608^R^ | 32^R^ |
| 15 |  | 0.5^S^ | 0.25^S^ | 0.064^S^ | 0.5^S^ | 0.25^S^ | 1^S^ | 0.5^S^ | 0.125^S^ | 256^R^ | 0.25^S^ | 0.008^S^ | 0.064/1.216^S^ | 0.064^S^ |
| SCV 15 |  | 0.5^S^ | 256^R^ | 0.125^S^ | 0.5^S^ | 0.5^S^ | 2^S^ | 1^S^ | 32^R^ | 256^R^ | 0.25^S^ | 32^R^ | 32/608^R^ | 0.064^S^ |
| 29 |  | 16^R^ | 256^R^ | 32^R^ | 32^R^ | 4^S^ | 2^S^ | 1^S^ | 32^R^ | 256^R^ | 256^R^ | 0.008^S^ | 0.032/0.608^S^ | 0.5^S^ |
| SCV 29 |  | 256^R^ | 256^R^ | 32^R^ | 32^R^ | 16^S^ | 4^S^ | 1^S^ | 32^R^ | 256^R^ | 256^R^ | 32^R^ | 32/608^R^ | 32^R^ |

MICs, minimum inhibitory concentrations; SCV, small colony variant; PEN, penicillin; OXA, oxacillin; CIP, ciprofloxacin; LEV, levofloxacin; GEN, gentamicin; AMI, amikacin; VAN, vancomycin; CLI, clindamycin; ERY, erythromycin; LNZ, linezolid; RIF, rifampicin; SXT, sulfamethoxazole-trimethoprim; TIG, tigecycline; ^R^, resistance; ^S^, susceptibility
