# Supplementary material for: Integrative omics analysis reveals insights into small colony variants of Staphylococcus aureus induced by sulfamethoxazole-trimethoprim
Source: BMC Microbiol. 2024 Jun 14;24:212. doi: 10.1186/s12866-024-03364-8 (PMC11179224; doi:10.1186/s12866-024-03364-8)
Supplement: Supplementary file 1 — Additional file 1: Table S1. Information of S. aureus used for the induction of SCVs. Table S2. Sequences of primers used for PCR in this study. Table S3. The minimal inhibitory concentrations of SCVs compared with those of the corresponding parental strains.Table S4. Transcriptic data. Table S5. The representative DEGs. Table S6. GO data. Table S7. KEGG data. Table S8. All differentially expressed ions in metabolomic analysis. Table S9. KEGG pathhways enriched by differentially expressed ions. [file 12866_2024_3364_MOESM1_ESM.zip › Table S5 The representative DEGs.docx]

**Table S5**. The top 30 most significantly DEGs in SCVs compared with its parental strain.

| **Gene ID** | **Gene name** | **Fold change** | **Product/Description** | **Definition** | **Pathway involved in** |
| --- | --- | --- | --- | --- | --- |
| NWMN_0176 | *ldh1* | -184.00 | L-lactate dehydrogenase | L-lactate dehydrogenase | Glycolysis/gluconeogenesis; cysteine and methionine metabolism; Pyruvate metabolism; propanoate metabolism; metabolic pathways; biosynthesis of secondary metabolites; microbial metabolism in diverse environments; biosynthesis of antibiotics |
| itsNWMN_0534 | *hxlB* | -167.33 | 6-phospho-3-hexuloisomerase | 6-phospho-3-hexuloisomerase | Pentose phosphate pathway; ethane metabolism; metabolic pathways; microbial metabolism in diverse environments; carbon metabolism; biosynthesis of amino acids |
| NWMN_2377 | NWMN_2377 | -70.44 | Hypothetical protein | Conserved hypothetical protein |  |
| NWMN_2268 | *lldP* | -49.41 | MarR family transcriptional regulator | L-lactate permease 2 |  |
| NWMN_0163 | *pflA* | -13.15 | pyruvate formate-lyase-activating protein | Pyruvate formate lyase activating enzyme |  |
| NWMN_2515 | *nrdD* | -11.77 | Anaerobic ribonucleoside-triphosphate reductase | Ribonucleoside-triphosphate reductase (formate) | Purine metabolism; pyrimidine metabolism |
| NWMN_0219 | *esxA* | -9.90 | WXG100 family type VII secretion effector EsxA | Conserved hypothetical protein |  |
| NWMN_0577 | *adhP* | -9.00 | Alcohol dehydrogenase AdhP | Alcohol dehydrogenase | Glycolysis/gluconeogenesis; fatty acid degradation; tyrosine metabolism; chloroalkane and chloroalkene degradation; naphthalene degradation; metabolic pathways; biosynthesis of secondary metabolites; microbial metabolism in diverse environments; biosynthesis of antibiotics; degradation of aromatic compounds |
| NWMN_0247 | *focA* | -8.88 | Formate/nitrite transporter family protein | Formate/nitrite transporter family protein |  |
| NWMN_0125 | *yagU* | -8.39 | YagU family protein | Putative membrane protein |  |
| NWMN_1831 | *ftnA* | -8.31 | H-type ferritin FtnA | Ferritin |  |
| NWMN_0162 | *pflB* | -8.12 | Formate acetyltransferase | Formate C-acetyltransferase" | Pyruvate metabolism; propanoate metabolism; butanoate metabolism; metabolic pathways; microbial metabolism in diverse environments |
| NWMN_0218 | NWMN_0218 | -7.43 | CHAP domain-containing protein | Staphyloxanthin biosynthesis protein |  |
| NWMN_2448 | *clpL* | -6.89 | ATP-dependent Clp protease ATP-binding subunit ClpL | ATP-dependent Clp protease ATP-binding subunit ClpL |  |
| NWMN_0175 | NWMN_0175 | -6.82 | FAD-binding oxidoreductase | Nitric oxide dioxygenase |  |
| NWMN_2514 | *nrdG* | -6.03 | Anaerobic ribonucleoside-triphosphate reductase activating protein | Anaerobic ribonucleoside-triphosphate reductase activating protein |  |
| NWMN_0366 | *yeaQ* | -6.02 | GlsB/YeaQ/YmgE family stress response membrane |  |  |
| NWMN_2087 | NWMN_2087 | -5.92 | DUF2273 domain-containing protein |  |  |
| NWMN_1999 | *sceD* | 18.28 | Lytic transglycosylase SceD |  |  |
| NWMN_2199 | NWMN_2199 | 9.67 | Secretory antigen precursor SsaA | CHAP domain-containing protein" |  |
| NWMN_1834 | NWMN_1834 | 9.56 | DUF3267 domain-containing protein | Putative zincin peptidase |  |
| NWMN_1249 | *guaC* | 8.13 | GMP reductase | Guanosine 5'-monophosphate oxidoreductase | Purine metabolism |
| NWMN_0542 | *vraX* | 7.59 | C1q-binding complement inhibitor VraX |  |  |
| NWMN_0845 | *clpB* | 7.32 | ATP-dependent chaperone ClpB | ATP-dependent Clp protease ATP-binding subunit ClpB |  |
| NWMN_0080 | *deoD* | 6.96 | Purine nucleoside phosphorylase | Purine-nucleoside phosphorylase | Purine metabolism; pyrimidine metabolism; nicotinate and nicotinamide metabolism; metabolic pathways; biosynthesis of secondary metabolites |
| NWMN_2509 | *betA* | 6.38 | Choline dehydrogenase | Choline dehydrogenase | Glycine, serine and threonine metabolism; metabolic pathways |
| NWMN_0210 | *lytM* | 6.21 | Lysostaphin | Glycine-glycine endopeptidase LytM |  |
| NWMN_2432 | NWMN_2432 | 6.15 | Hypothetical protein |  |  |
| NWMN_2266 | NWMN_2266 | 6.05 | DUF3021 domain-containing protein |  |  |
| NWMN_1938 | *groES* | 5.93 | Chaperonin GroES | Chaperonin GroES |  |
