# Supplementary material for: Integrative omics analysis reveals insights into small colony variants of Staphylococcus aureus induced by sulfamethoxazole-trimethoprim
Source: BMC Microbiol. 2024 Jun 14;24:212. doi: 10.1186/s12866-024-03364-8 (PMC11179224; doi:10.1186/s12866-024-03364-8)
Supplement: Supplementary file 2 — Additional file 2: Figure S1. aureus SCV (strain 15, 2 and 29) on Mueller–Hinton (MH) agar with thymidine, menadione, or hemin. The sizes of the SCV colonies on MH plates with thymidine, menadione, or hemin were similar to those on MH plates without these compounds. This suggests that the SCVs were independent of thymidine, menadione, or hemin. A-D SCV 15. E-H SCV 2. I-L SCV 29. A, E, I Control without any compounds. B, F, J Thymidine. C, G, K Menadione. D, H, L Hemin. Figure S2. Growth curves of three pairs of S. aureus strains and their corresponding SCVs in tryptic soy broth (TSB). The curves clearly demonstrate that the growth of the SCVs was significantly delayed when compared to their corresponding parental strains. Figure S3. Phylogenetic tree depicting the relationship between S. aureus strain 15, its corresponding SCV, and other S. aureus strains. Through phylogenetic analysis, we discovered a strong association between strain 15 and its SCV with S. aureus strains USA300, COL, and NCTC 8325. Figure S4. Mauve alignments showcasing the genomes of S. aureus NCTC 8325, strain 15, and SCV 15. Each colored region represents a locally collinear block (LCB) where no rearrangement of homologous backbone sequences is observed. Mauve analysis revealed a few genomic rearrangement events in the SCV strain compared to the parental strain. Figure S5. Correlation heatmap illustrating the association between DEGs and SDMs enriched in KEGG pathways. The Spearman method was employed for correlation analysis. A higher number of "*" indicates a smaller p-value. The intensity of color and the quantity of "*" within each square reflect the significance of the association between the gene and the metabolite. DEGs, differentially expressed genes; SDMs, significantly different metabolites. [file 12866_2024_3364_MOESM2_ESM.pdf]

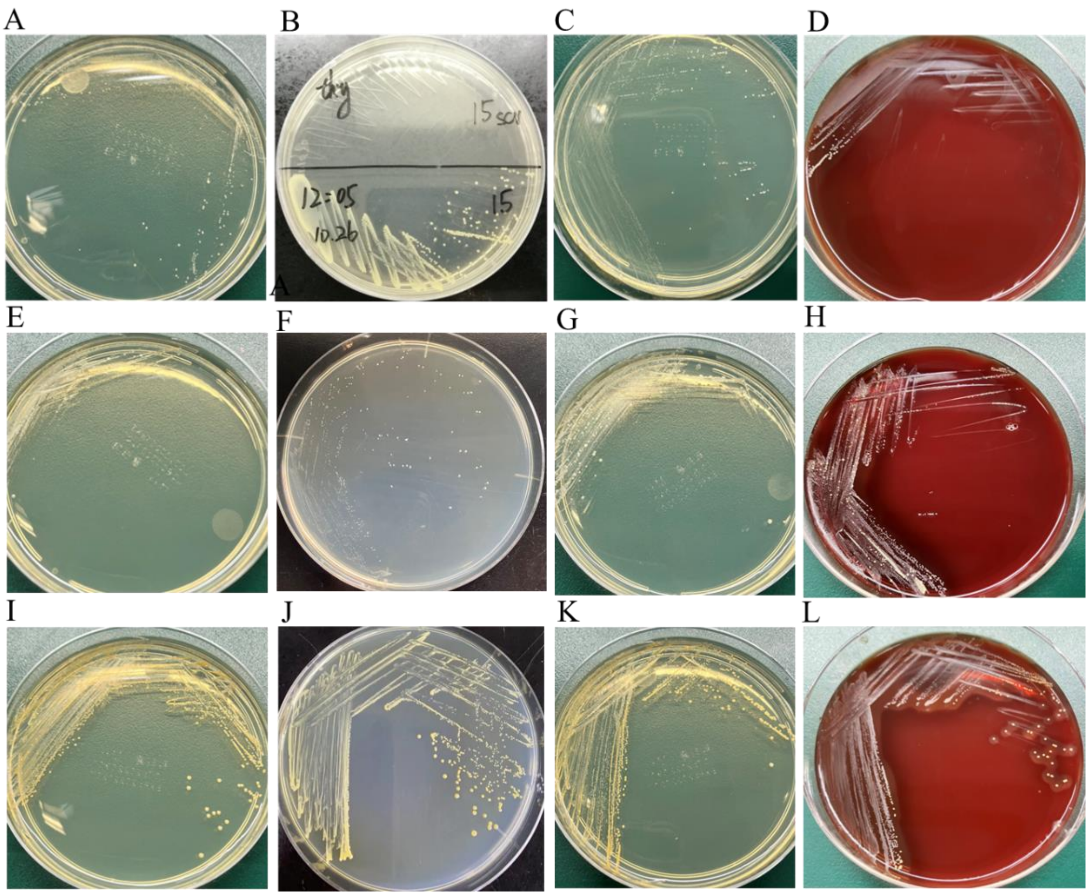

Figure S1: *S. aureus* SCV (strain 15, 2 and 29) on Mueller–Hinton (MH) agar with thymidine, menadione, or hemin. The sizes of the SCV colonies on MH plates with thymidine, menadione, or hemin were similar to those on MH plates without these compounds. This suggests that the SCVs were independent of thymidine, menadione, or hemin. A-D SCV 15. E-H SCV 2. I-L SCV 29. A, E, I Control without any compounds. B, F, J Thymidine. C, G, K Menadione. D, H, L Hemin

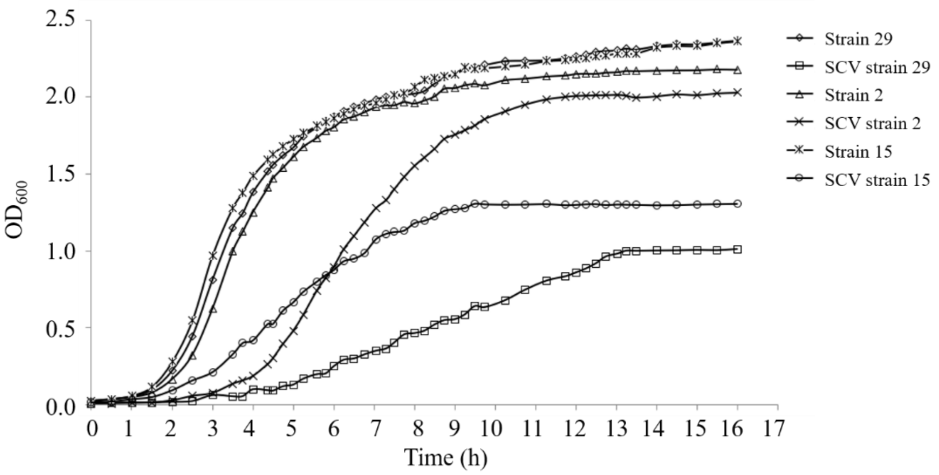

Figure S2: Growth curves of three pairs of *S. aureus* strains and their corresponding SCVs in tryptic soy broth (TSB). The curves clearly demonstrate that the growth of the SCVs was significantly delayed when compared to their corresponding parental strains

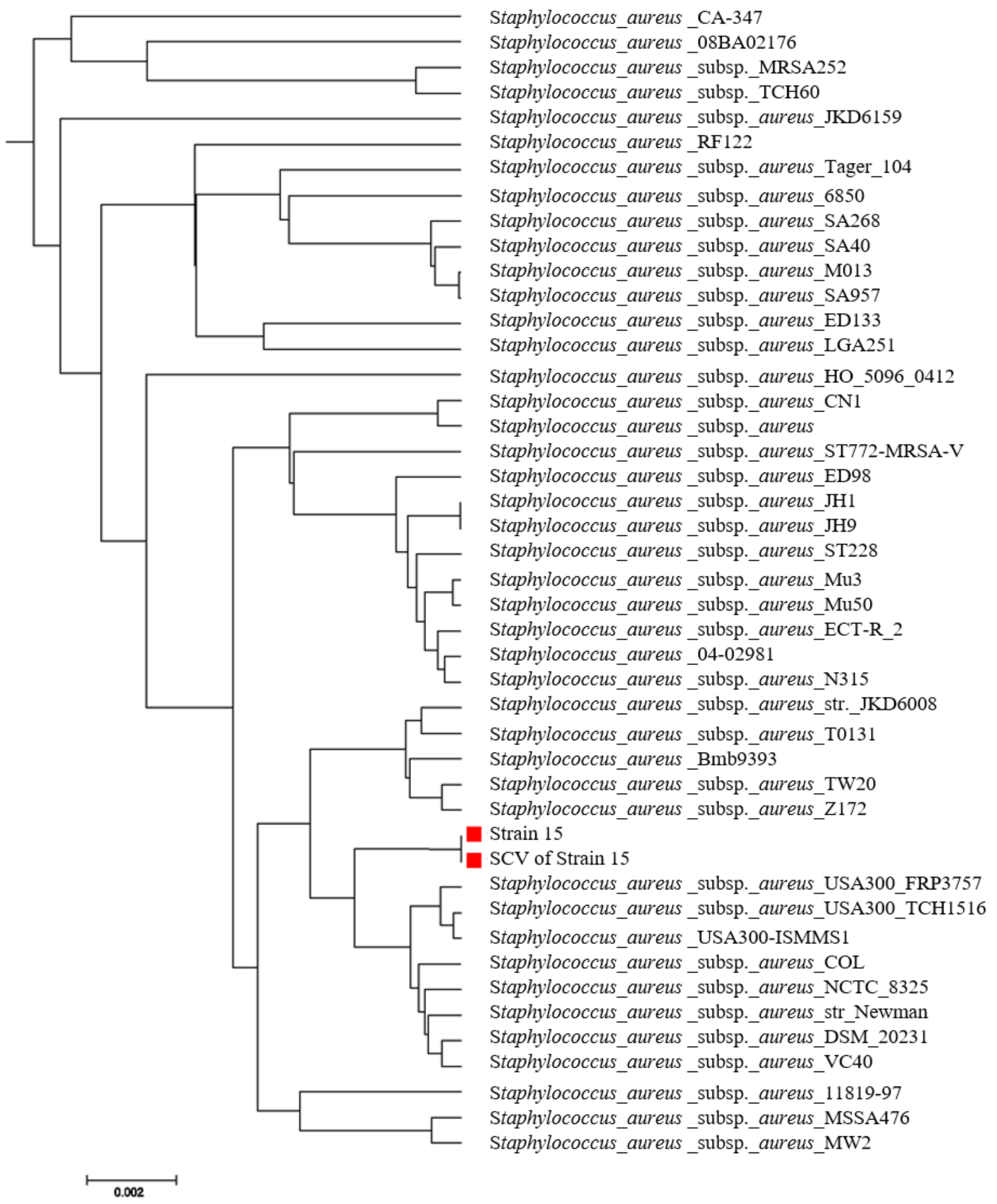

Figure S3: Phylogenetic tree depicting the relationship between *S. aureus* strain 15, its corresponding SCV, and other *S. aureus* strains. Through phylogenetic analysis, we discovered a strong association between strain 15 and its SCV with *S. aureus* strains USA300, COL, and NCTC 8325

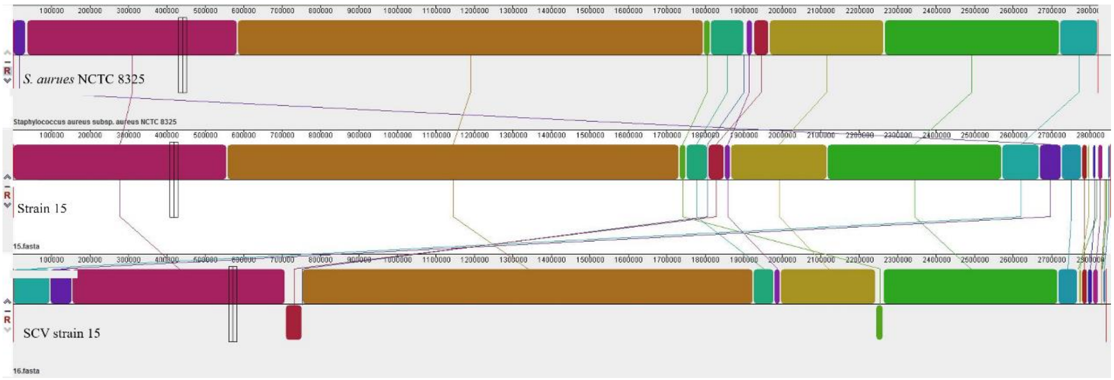

Fig S4 Mauve alignments showcasing the genomes of *S. aureus* NCTC 8325, strain 15, and SCV strain 15. Each colored region represents a locally collinear block (LCB) where no rearrangement of homologous backbone sequences is observed. Mauve analysis revealed a few genomic rearrangement events in the SCV strain compared to the parental strain

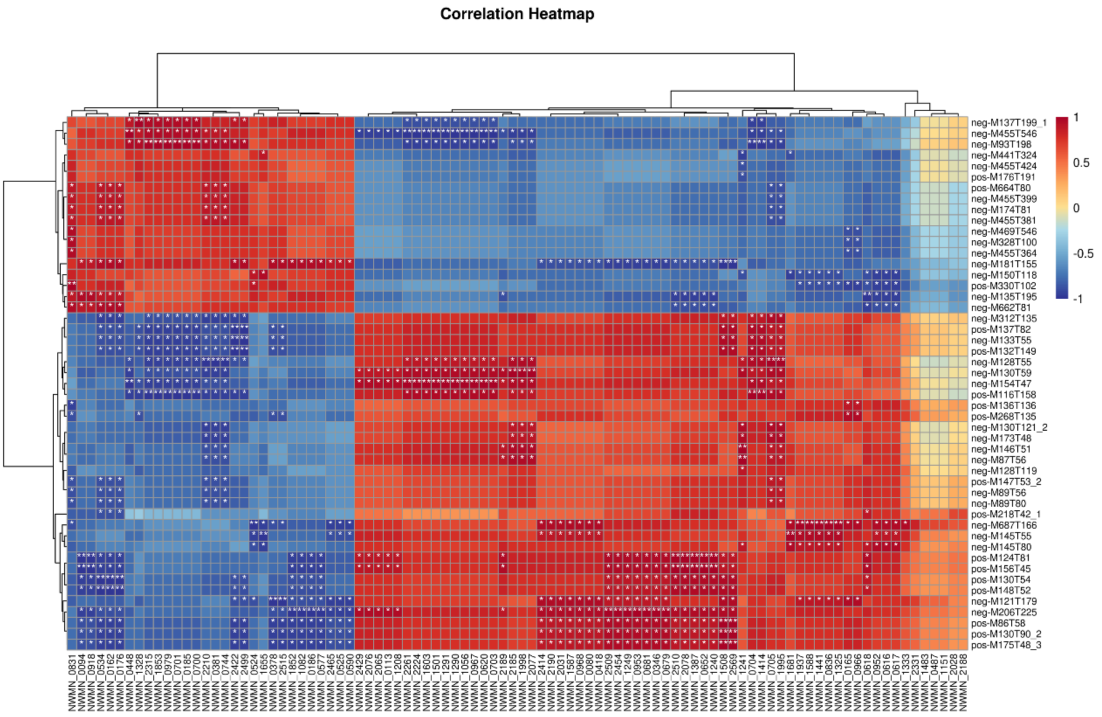

Fig S5 Correlation heatmap illustrating the association between DEGs and SDMs enriched in KEGG pathways. The Spearman method was employed for correlation

analysis. A higher number of "*" indicates a smaller p-value. The intensity of color and the quantity of "*" within each square reflect the significance of the association between the gene and the metabolite. DEGs, differentially expressed genes; SDMs, significantly different metabolites
