# Supplementary figures and images for: Integrative omics analysis reveals insights into small colony variants of Staphylococcus aureus induced by sulfamethoxazole-trimethoprim
Source: BMC Microbiol. 2024 Jun 14;24:212. doi: 10.1186/s12866-024-03364-8 (PMC11179224; doi:10.1186/s12866-024-03364-8)

Marker

29 29scv

15 15scv

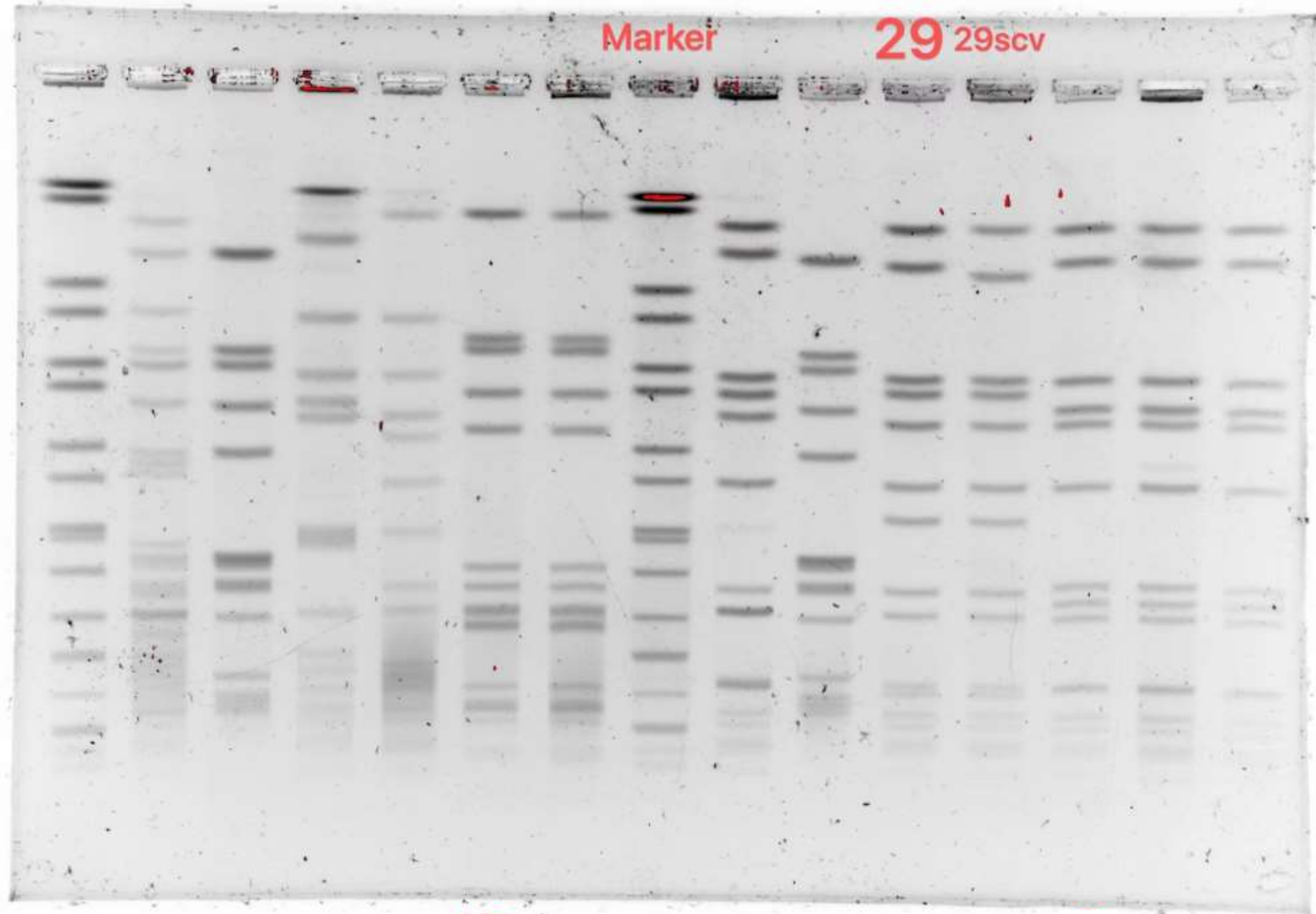

Marker

28scv

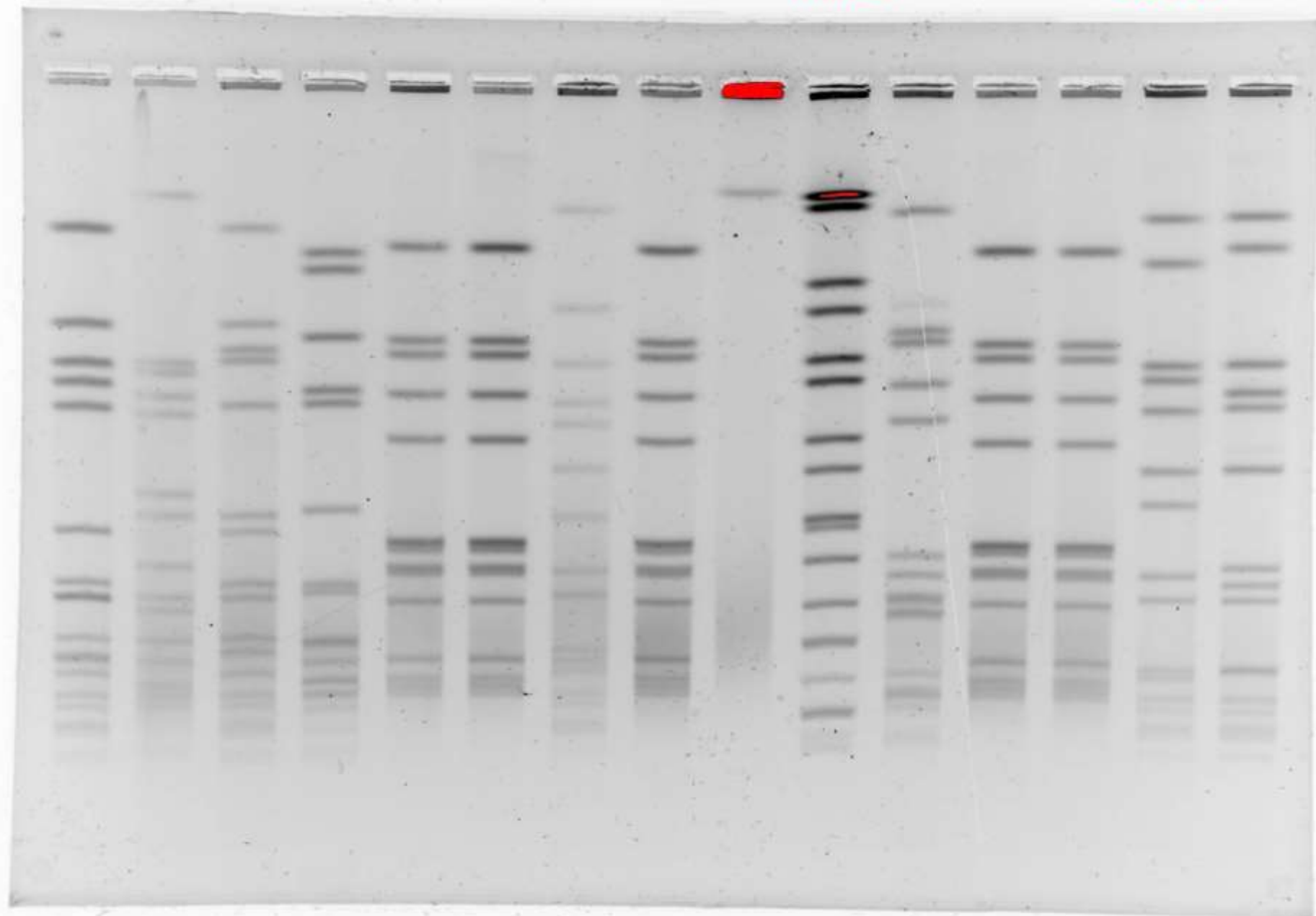

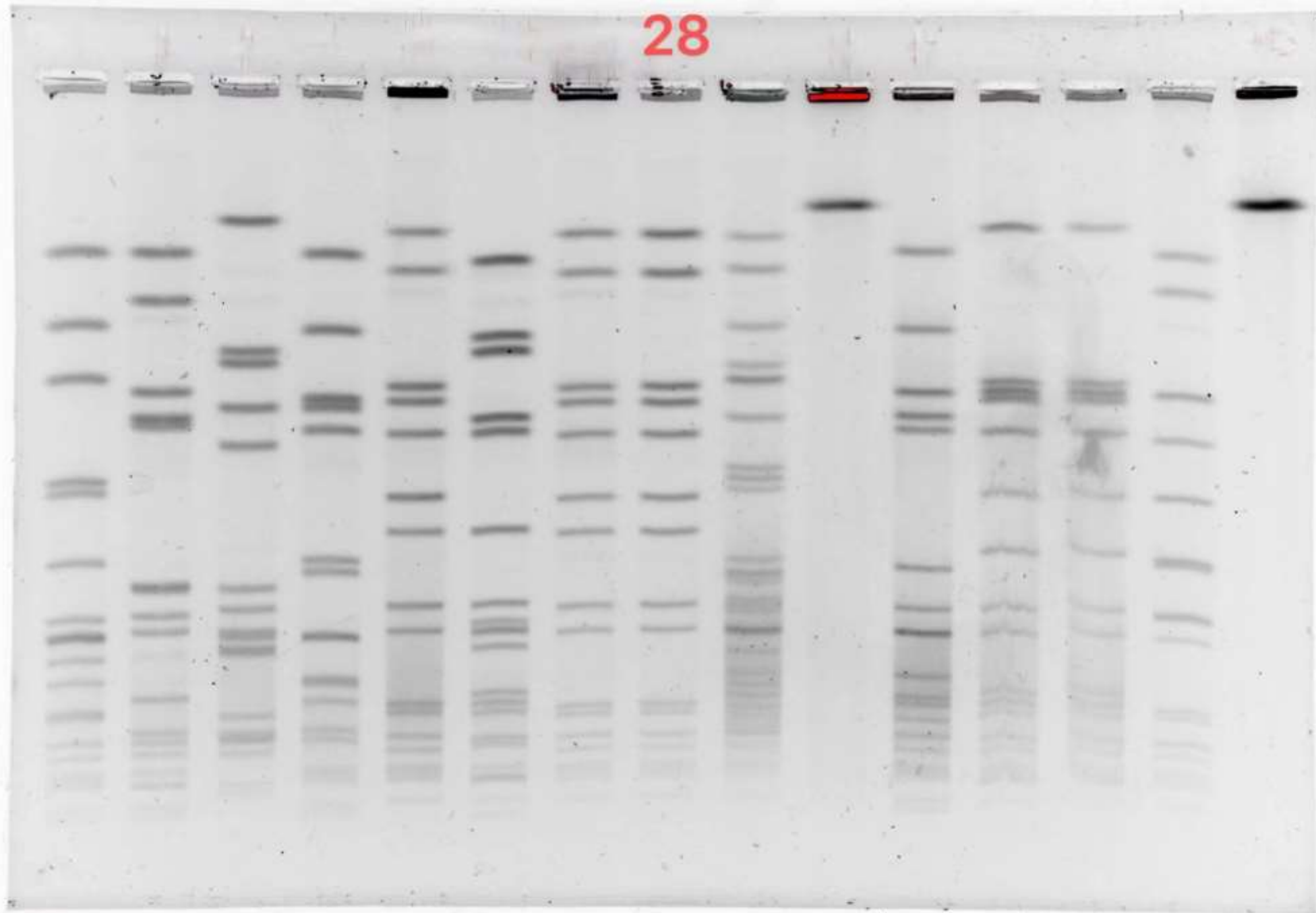

Supplement: Supplementary file 3 — Additional file 3. [file 12866_2024_3364_MOESM3_ESM.pdf]
